# *BICD1* expression, as a potential biomarker for prognosis and predicting response to therapy in patients with glioblastomas

**DOI:** 10.18632/oncotarget.22667

**Published:** 2017-11-27

**Authors:** Shang-Pen Huang, Yu-Chan Chang, Qie Hua Low, Alexander T.H. Wu, Chi-Long Chen, Yuan-Feng Lin, Michael Hsiao

**Affiliations:** ^1^ Graduate Institute of Clinical Medicine, College of Medicine, Taipei Medical University, Taipei, Taiwan; ^2^ Department of Neurology, PoJen General Hospital, Taipei, Taiwan; ^3^ Genomics Research Center, Academia Sinica, Taipei, Taiwan; ^4^ Yong Loo Lin School of Medicine, National University of Singapore, Singapore, Singapore; ^5^ The Ph.D. Program for Translational Medicine, College of Medical Science and Technology, Taipei Medical University and Academia Sinica, Taipei, Taiwan; ^6^ Department of Pathology, College of Medicine, Taipei Medical University, Taipei, Taiwan; ^7^ Department of Pathology, Taipei Medical University Hospital, Taipei, Taiwan; ^8^ Department of Biochemistry, College of Medicine, Kaohsiung Medical University, Kaohsiung, Taiwan

**Keywords:** BICD1, glioblastoma (GBM), temozolomide (TMZ), biomarker, MGMT

## Abstract

There is variation in the survival and therapeutic outcome of patients with glioblastomas (GBMs). Therapy resistance is an important challenge in the treatment of GBM patients. The aim of this study was to identify Temozolomide (TMZ) related genes and confirm their clinical relevance. The TMZ-related genes were discovered by analysis of the gene-expression profiling in our cell-based microarray. Their clinical relevance was verified by *in silico* meta-analysis of the Cancer Genome Atlas (TCGA) and the Chinese Glioma Genome Atlas (CGGA) datasets. Our results demonstrated that *BICD1* expression could predict both prognosis and response to therapy in GBM patients. First, high *BICD1* expression was correlated with poor prognosis in the TCGA GBM cohort (n=523) and in the CGGA glioma cohort (n=220). Second, high *BICD1* expression predicted poor outcome in patients with TMZ treatment (n=301) and radiation therapy (n=405). Third, multivariable Cox regression analysis confirmed *BICD1* expression as an independent factor affecting the prognosis and therapeutic response of TMZ and radiation in GBM patients. Additionally, age, *MGMT* and *BICD1* expression were combinedly utilized to stratify GBM patients into more distinct risk groups, which may provide better outcome assessment. Finally, we observed a strong correlation between *BICD1* expression and epithelial-mesenchymal transition (EMT) in GBMs, and proposed a possible mechanism of *BICD1*-associated survival or therapeutic resistance in GBMs accordingly. In conclusion, our study suggests that high *BICD1* expression may result in worse prognosis and could be a predictor of poor response to TMZ and radiation therapies in GBM patients.

## INTRODUCTION

Gliomas, the most common neoplasms found in primary brain tumors, are graded according to histologic subtype [1]. WHO (World Health Organization) defines glioblastomas (GBMs) as grade IV gliomas, which are the most common primary brain tumor in adults [2]. The prognosis of patients with GBMs is very poor. The mean survival is only from 10 to 14 months [3, 4], indicating variable clinical behavior and response to therapy.

GBMs are difficult to treat for several reasons. First, a large number of drugs cannot get into tumor sites directly due to the existence of the blood-brain barrier (BBB). Second, since GBMs can infiltrate the surrounding tissues, the standard therapeutic regimens, including surgery, radiation, and chemotherapy, cannot remove them completely. Finally, chemotherapy drugs, which are specific to one tumor cell type, cannot kill all tumor cells due to their heterogeneity.

Temozolomide (TMZ), an alkylating agent prodrug, is an orally administered chemotherapy with a good penetration of the BBB and limited side effects. It is the only US FDA-approved drug for treating refractory anaplastic astrocytomas in adult patients since 1999 [5], and newly diagnosed GBMs in adult patients since 2005 [6]. Its active form can methylate DNA at the sites of O^6^-guanine, N^7^-guanine, or N^3^-adenine. The DNA damage is primarily mediated by the O^6^-methylguanine (O^6^-MeG), which induces double-strand breaks and base mispairing, and thereby causes apoptosis and cell death [7, 8]. Currently, radiotherapy with concomitant and adjuvant TMZ is the gold standard for patients with newly diagnosed GBMs [9]. Although the combined use of TMZ and radiation have improved patients’ survival more than radiation alone (median survival from 12.1 to 14.6 months in normal population [10], and median survival from 7.6 to 9.3 months in elder population [11]), almost all patients experience tumor progression or recurrence. Local tumor progression is the predominant pattern of treatment failure [12]. In general, tumor recurrence is associated with poor survival because treatment options are limited [13].

Therapeutic resistance is a critical factor affecting the survival rate of cancer patients. TMZ and radiation resistance are two major issues in the management of GBMs. The existing mechanisms of DNA repair in GBM cells limit the cytotoxic effect of TMZ in treatment of GBMs [14, 15]. Moreover, the results obtained from studies of intrinsic and acquired TMZ resistance in GBM cells support the idea that TMZ resistance is not mediated by only a single molecular event, but by multiple ones. Therefore, exploring the possible mechanisms of therapeutic resistance within GBM cells is an important mission of neuro-oncologists, and identification of biomarkers that are associated with therapeutic resistance in GBMs might provide a feasible way for pursuing this goal.

Recent advances in the development of molecular markers by genome-wide studies of the CNS tumors have improved our understanding of the biology in these tumors. To date, however, only a few molecular markers really have clinical relevance in the therapeutic decision-making of GBM patients. *MGMT* promoter methylation in high-grade astrocytomas and co-deletion of 1p/19q in oligodendrogliomas are proven prognostic and predictive markers that play a role in standard practice, and mutations of *IDH1* or *IDH2* are of strong prognostic value in lower grade gliomas (LGG), which are the most widely validated biomarkers in neuro-oncology currently [16, 17].

Although the promoter methylation status of *MGMT* is shown to be a useful prognostic or predicting biomarker in the elderly patients with newly diagnosed GBM [18, 19], the role of *MGMT* in clinical decision-making remains limited and the routine analysis of the *MGMT* promoter methylation status is restricted to only a few clinical scenarios. Although various testing methods, including methylation-specific polymerase chain reaction (MS-PCR), pyrosequencing, methylation-specific multiplex ligation-dependent probe amplification, and immunohistochemistry (IHC), are currently being used, however, there is still no uniform methodology for the *MGMT* testing. Therefore, standardized procedures should be created to allow inter-laboratory reproducibility, especially if future treatment decisions will be based on these results [20]. In fact, the IHC analysis of MGMT protein lacks a significant correlation with the *MGMT* promoter methylation status, and is not routinely used for diagnostic purposes due to the inter-observer variability [21]. Thus, there is an urgent need to identify a specific and sensitive biomarker for prognosis and predicting the response to therapy, which may provide better therapeutic guide in the management of glioma patients [22–24].

In this study, we identified *BICD1* expression as a potential biomarker from cell-based microarray data, and validated its prognostic value in clinical datasets of the TCGA GBM and GBMLGG cohorts, and the CCGA glioma cohort. BICD Cargo Adaptor 1 (*BICD1*), is a human homologue of the Drosophila Bicaudal-D gene [25]. This is the first report to investigate the association of *BICD1* expression with the prognosis and therapeutic outcome of GBM patients. Our results confirmed our hypothesis that *BICD1* expression is a potential biomarker for prognosis and predicting the response to therapy in patients with GBMs.

## RESULTS

### An overview of the overall survival of patients in the TCGA GBMLGG (glioblastoma and lower grade glioma) cohort

From the TCGA GBMLGG cohort data of patients with gliomas (http://www.xenabrowser.net/), the lower grade gliomas (grades II and III gliomas, LGGs) were the less malignant phenotype. The median survival time was 7.29±0.86 years and 5-year survival rate was 61.5% in patients with LGGs. The grade IV gliomas (Glioblastomas, GBMs) were the most malignant phenotype in all gliomas. The median survival time was only 1.13±0.07 years and 2-year survival rate was 20.9% in patients with GBMs. The median survival time was 4.09±0.44 years and 5-year survival rate was 45.7% in all glioma patients (Table 1).

**Table 1:** The overall survival of patients with different histological grade in the TCGA GBMLGG cohort

Histological grade	Patient numbers	Death event number	Median value				5-year survival rate	2-year survival rate
			Survival time (years)	S.D.	Lower limit (95% CI)	Upper limit (95% CI)		
**LGG (Grades II+III)**	524	133	7.29	0.86	5.6	8.98	61.5%	85.6%
**GBM (Grade IV)**	165	127	1.13	0.07	1.00	1.27	0%	20.9%
**All (Grades II+III+IV)**	689	260	4.09	0.44	3.22	4.96	45.7%	69.8%

### Discovery of candidate markers by analyzing the TMZ-related genomic alterations in GBM cell lines and validating their prognostic values in the TCGA GBM database

**Figure 1:**
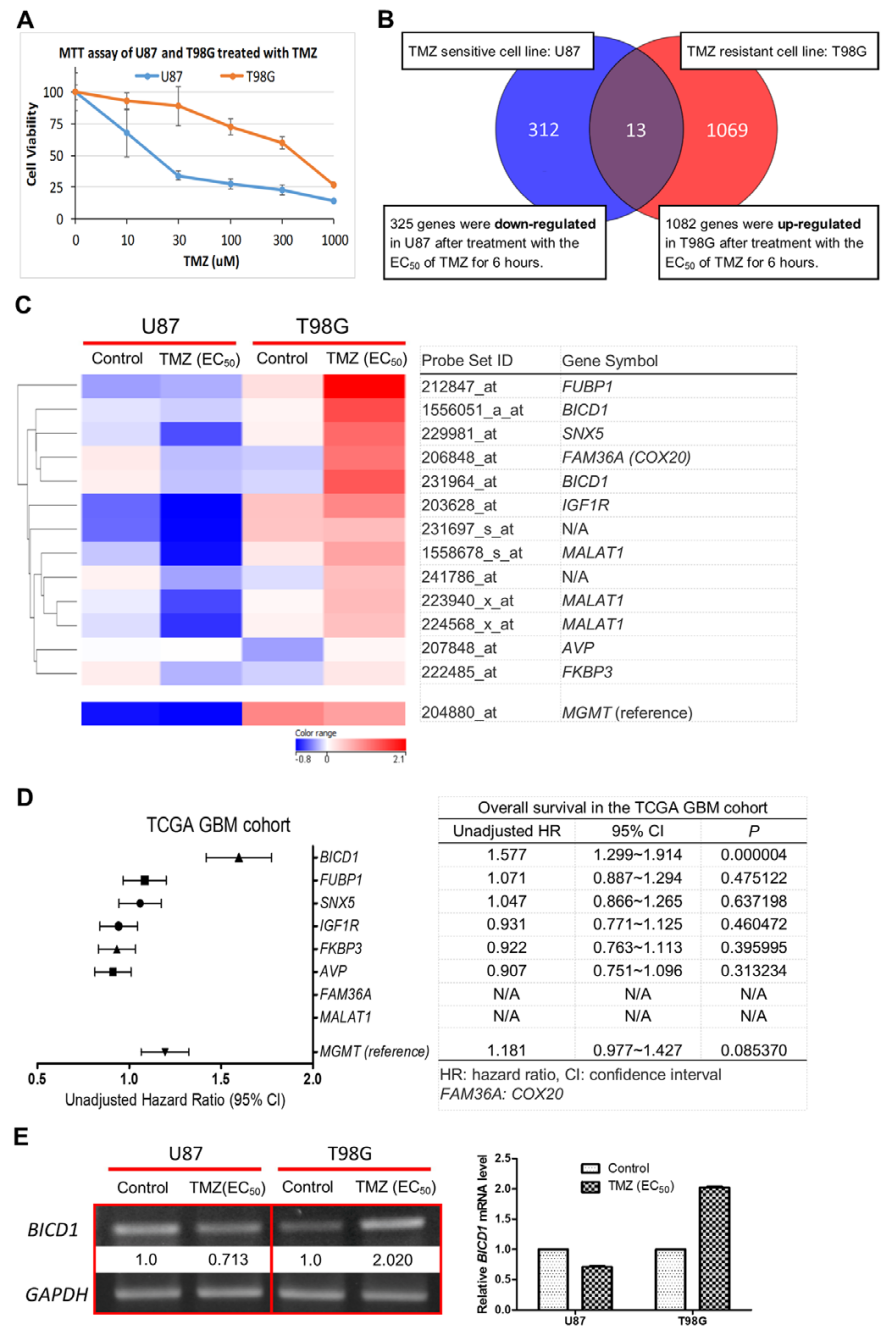
Identification of *BICD1* expression as a potential biomarker of GBMs **(A)** Results of MTT assay showed a higher cell viability in T98G than in U87 when both cell lines were treated with the alkylating agent TMZ. The EC_50_ of TMZ was 400μM in T98G and the EC_50_ of TMZ was 20μM in U87. **(B)** Hierarchical clustering analysis of the genomic alterations in T98G (TMZ resistant cell line) and in U87 (TMZ sensitive cell line) after treatment with their EC_50_ of TMZ for 6 hours. A total of 13 probes which were up-regulated in T98G and down-regulated in U87 after TMZ treatment were identified. **(C)** A heat map for identification of potential candidate genes was constructed according to the extent of gene expression change after TMZ treatment in T98G and U87. A total of 8 candidate genes were identified (2 probes lacked gene symbol; *BICD1* was represented by 2 probes; *MAMAT1* was represented by 3 probes). **(D)** The prognostic values of these candidate genes were verified and compared using the Kaplan-Meier survival analysis of the clinical dataset in the TCGA GBM cohort. The unadjusted hazard ratio (HR), upper and lower 95% confidence interval (CI), and *P* value, which were determined by the expression status of each candidate gene, were ranked and listed according to their HR. **(E)** RT-PCR was performed to confirm the gene expression change of *BICD1* in GBM cell line after TMZ treatment in our microarray data. Results of RT-PCR showed increased *BICD1* mRNA expression in T98G cells after TMZ treatment but decreased *BICD1* mRNA expression in U87 cells after TMZ treatment (The percentage of brightness and contrast had been adjusted to increase the *BICD1* mRNA signal. The percentage of 65% in brightness and 80% in contrast were applied in the presentation of *BICD1* mRNA expression). The gene expression status of *MGMT* and its prognostic value are shown in (C) and (D) as reference.

Biomarkers have been developed through several ways, including cell line models [26]. U87 and T98G are two well-known GBM cell lines [27, 28]. U87 is sensitive to TMZ, but T98G is resistant to it. The EC_50_ of TMZ in U87 and T98G have been well studied by a large number of research groups [29–32]. According to our result of MTT assay, the EC_50_ of TMZ was 400μM in T98G, and 20μM in U87 (Figure 1A), which were compatible with other study groups’ data. In this study, U87 and T98G cells were treated with their EC_50_ of TMZ for 6 hours and the genomic alterations in each cell line were observed using gene expression microarrays. A total of 13 probes, which were up-regulated in T98G and down-regulated in U87 after TMZ treatment, were identified by the method of hierarchical clustering analysis (Figure 1B). The expression changes of these probes in GBM cells under different conditions (control vs. EC_50_ of TMZ) were analyzed using the differential expression analysis of our microarray data. A heat map was constructed by ranking these probes according to the extent of expression change in T98G and U87 (with or without TMZ treatment), and there were 8 genes represented by these 13 probes (Figure 1C). The prognostic values of the 8 genes were then verified by the Kaplan-Meier survival analysis of the clinical dataset in the TCGA GBM cohort. The unadjusted hazard ratio (HR), upper and lower 95% confidence interval (CI), and *P* value, which were determined by the expression status of the 8 genes, were calculated and compared. And the 8 candidate genes were ranked according to their HR (Figure 1D). Notably, *FUBP1* expression was highly increased in T98G after TMZ treatment and was the top-ranked marker out of the 13 identified probes (Figure 1C). However, when examining its prognostic value, *FUBP1* expression was shown to have no significance in predicting the overall survival of GBM patients (HR=1.071; 95% CI=0.887-1.294; *P=*0.475122) (Figure 1D). This was inconsistent with the past research whereby *FUBP1* was shown to be associated with poor prognosis in glioma patients [33]. Therefore, we chose *BICD1,* the top-ranked gene in impacting the overall survival of GBM patients (HR=1.577; 95% CI=1.299-1.914; *P=*0.000004) (Figure 1D), as a candidate marker because of its high potential in developing a biomarker of GBMs, and its novelty in the study of GBMs. The differential expression of *BICD1* mRNA in U87 and T98G (with or without TMZ treatment) was further confirmed by RT-PCR (Figure 1E). Additionally, the gene expression status of *MGMT* in U87 and T98G (with or without TMZ treatment) and its prognostic value were analyzed and presented as reference (Figure 1C, 1D).

### *BICD1* expression was significantly correlated with the WHO grade, patient age, and KPS in the TCGA GBMLGG cohort, and highly associated with the molecular subclassification of GBMs

**Figure 2:**
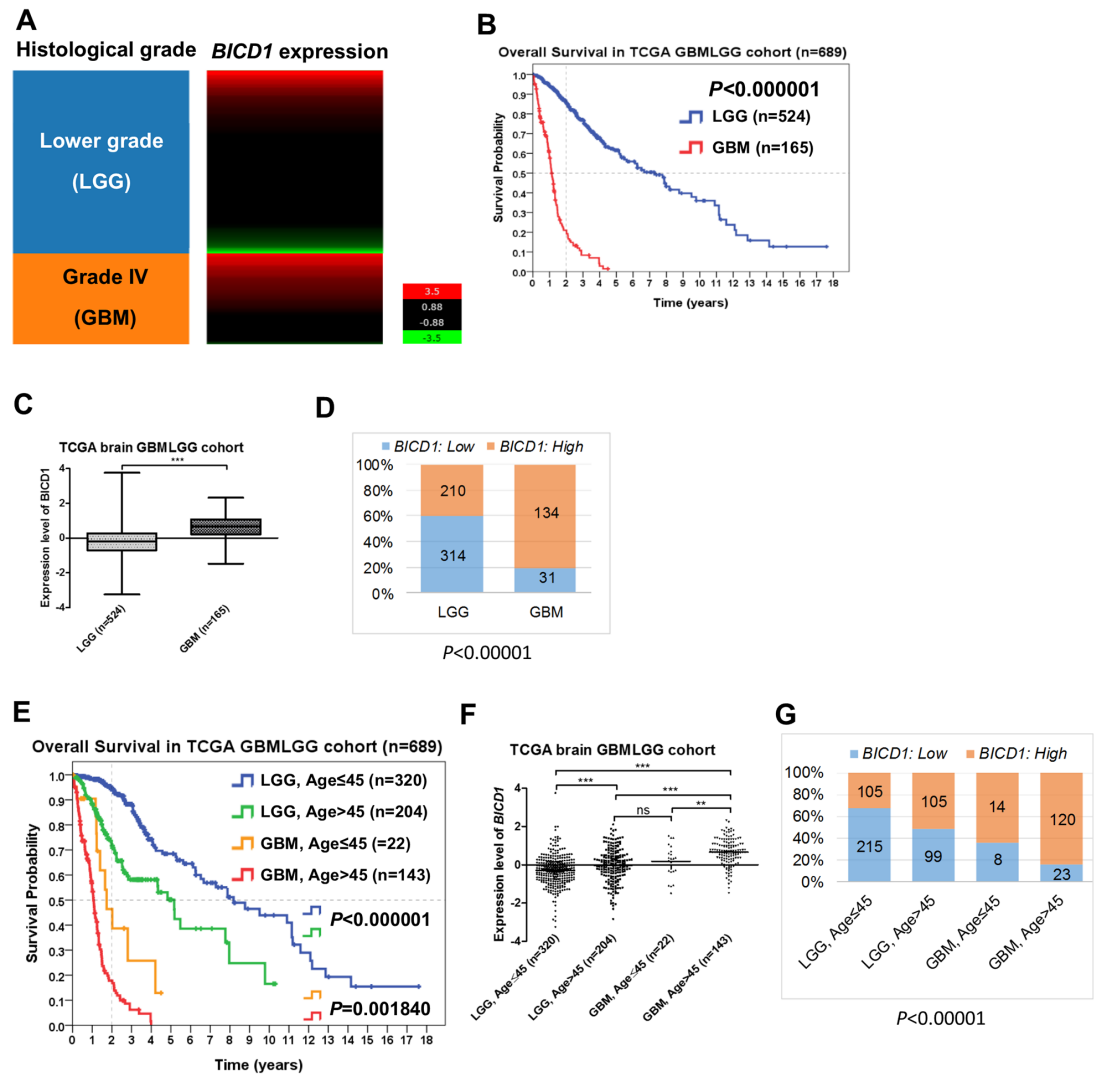
The expression status of *BICD1* in different histological grade and age group obtained by the gene expression RNAseq (polyA+ IlluminaHiSeq) in the TCGA GBMLGG cohort (n=689) **(A)** Grade IV gliomas (GBMs) appeared to have higher *BICD1* expression than lower grade gliomas (LGGs). **(B)** GBM patients had significantly poorer overall survival than LGG patients (*P<*0.000001). **(C)** The expression levels of *BICD1* were significantly higher in GBMs than in LGGs (^***^). **(D)** GBMs had a significantly higher percentage of high *BICD1* expression than LGGs (GBMs: 134/165 vs. LGGs: 210/524, *P*<0.00001). **(E)** LGG and GBM patients in the TCGA GBMLGG cohort were further separated by age (≤45 vs. >45). Patients with higher age (>45) had significantly poorer overall survival in both LGG and GBM patient groups. The LGG patients with age>45 had significantly poorer overall survival than those with age≤45 (*P<*0.000001). The GBM patients with age>45 also had significantly poorer overall survival than those with age≤45 (*P=*0.001840). **(F)** The expression levels of *BICD1* were significantly higher in GBM patients with age>45, than in those with age≤45 (^**^). The expression levels of *BICD1* were also significantly higher in LGG patients with age>45 than in those with age≤45 (^***^). **(G)** The GBM patient group with age>45 had the highest percentage of high *BICD1* expression (120/143), but the LGG patient group with age≤45 had the lowest one (105/320). The percentage of high *BICD1* expression was significantly correlated with the poor prognosis of glioma patients when they were grouped according to the histological grade and patient age in the TCGA GBMLGG cohort (*P<*0.00001).

GBMs (grade IV gliomas) had apparently higher *BICD1* expression than LGGs (grades II and III gliomas) in the TCGA GBMLGG cohort (n=689) (Figure 2A). GBM patients had significantly poorer overall survival than LGG patients (*P<*0.000001) (Figure 2B). The expression levels of *BICD1* were significantly higher in GBMs than in LGGs (^***^) (Figure 2C). GBMs had a significantly higher percentage of high *BICD1* expression than LGGs (GBMs: 134/165 vs. LGGs: 210/524, *P<*0.00001) (Figure 2D) (Table 2).

**Table 2:** Correlation of *BICD1* expression with the clinicopathological features of patients in the TCGA GBMLGG cohort

Clinicopathological features	n	*BICD1* expression, n (%)		*P*
	689	Low, n=345 (50.1)	High, n=344 (49.9)	
**Age**				**<0.000001**
≤45	342	223 (65.2)	119 (34.8)	
>45	347	122 (35.2)	225 (64.8)	
**Gender**				0.724838
Female	295	150 (50.8)	145 (49.2)	
Male	394	195 (49.5)	199 (50.5)	
**WHO Grade**				**<0.000001**
LGG (Grades II+III)	524	314 (59.9)	210 (40.1)	
GBM (Grade IV)	165	31 (18.8)	134 (81.2)	

*BICD1* expression was highly and significantly correlated with the WHO histological grade (*P<*0.000001) and patient age (*P<*0.000001) in the TCGA GBMLGG cohort (Table 2). LGG and GBM patients in the TCGA GBMLGG cohort were further separated by age (≤45 vs. >45). Undoubtedly, patients with higher age had significantly poorer overall survival in both LGG and GBM patient groups in the TCGA GBMLGG cohort (Figure 2E). The LGG patients with age>45 had significantly poorer overall survival than those with age≤45 (*P<*0.000001). The GBM patients with age>45 also had significantly poorer overall survival than those with age≤45 (*P=*0.001840). The expression levels of *BICD1* were significantly higher in GBM patients with age>45 than in those with age≤45 (^**^). The expression levels of *BICD1* were also significantly higher in LGG patients with age>45 than in those with age≤45 (^***^) (Figure 2F). The GBM patients with age>45 had the highest percentage of high *BICD1* expression (120/143), and the LGG patients with age≤45 had the lowest percentage of high *BICD1* expression (105/320). The percentage of high *BICD1* expression was significantly correlated with poor prognosis in glioma patients when they are grouped according to the WHO grade and patient age in the TCGA GBMLGG cohort (*P<*0.00001) (Figure 2G).

*BICD1* expression was significantly but negatively correlated with patients’ clinical performance (Karnofsky performance score, KPS) in the TCGA GBMLGG cohort (≥90 vs. <90, *P<*0.000001) (Table 3). However, it was not significantly correlated with the KPS (≥90 vs. <90, *P*=0.609816) (Supplementary Table 1), and the clinicopathological features, including patient age (<65 vs. ≥65, *P*=0.089722), gender (Female vs. Male, *P*=0.136863), *MGMT* expression (Low vs. High, *P*=0.204737), and overall survival indicator (censor vs. death, *P*=0.712211), in the TCGA GBM cohort (Table 4).

**Table 3:** Correlation of *BICD1* expression with the KPS of patients in the TCGA GBMLGG cohort

Clinicopathological feature	n	*BICD1* expression, n (%)		*P*
	439	Low, n=220 (50.1)	High, n=219 (49.9)	
**KPS**				**<0.000001**
≥90	226	147 (65)	79 (35)	
<90	213	73 (34.3)	140 (65.7)	

KPS: Karnofsky performance score.

**Table 4:** Correlation of *BICD1* expression with the clinicopathological features of patients in the TCGA GBM cohort

Clinicopathological features	n	*BICD1* expression, n (%)		*P*
	523	Low, n=262 (50.1)	High, n=261 (49.9)	
**Age**				0.089722
<65	347	183 (52.7)	164 (47.3)	
≥65	176	79 (44.9)	97 (55.1)	
**Gender**				0.136863
Female	205	111 (54.1)	94 (45.9)	
Male	318	151 (47.5)	167 (52.5)	
***MGMT* expression**				0.204737
Low	262	124 (47.3)	138 (52.7)	
High	261	138 (52.9)	123 (47.1)	
**Overall survival indicator**				0.712211
0 (censor)	89	43 (48.3)	46 (51.7)	
1 (death)	434	219 (50.5)	215 (49.5)	
**Molecular Subclassification**				
Neural	87	70 (80.5)	17 (19.5)	**<0.00001**
Proneural	138	91 (65.9)	47 (34.1)	
Mesenchymal	156	71 (45.5)	85 (54.5)	
Classical	142	30 (21.1)	112 (78.9)	

**Figure 3:**
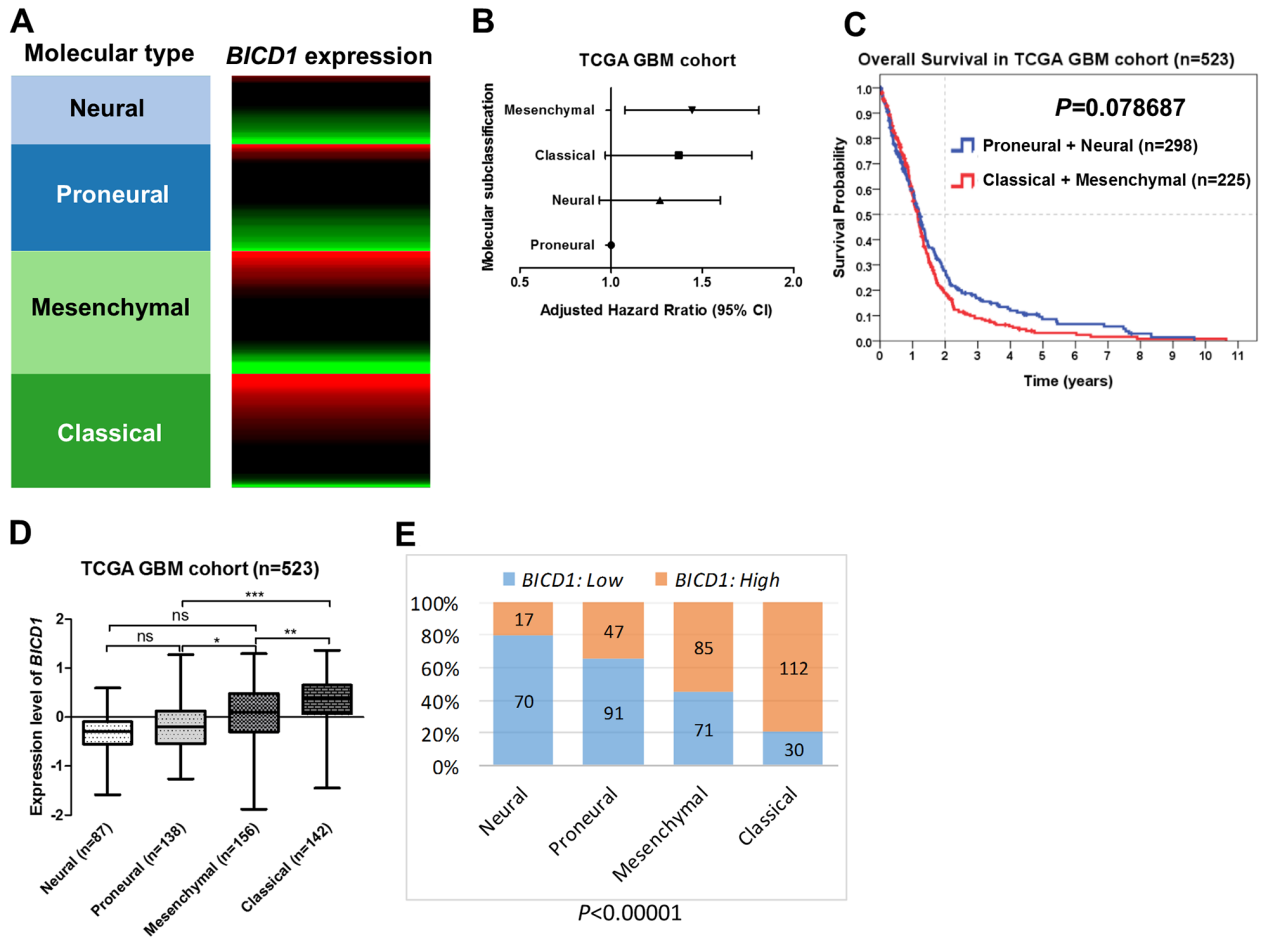
The expression status of *BICD1* in different molecular subclassification of GBMs obtained by the gene expression array (AffyU133a) in the TCGA GBM cohort (n=523) The molecular classification of GBMs was defined by TCGA. **(A)** *BICD1* expression varied with the molecular subclassification of GBMs, and appeared to be up-regulated in classical and mesenchymal subtypes. **(B)** The molecular subtypes of GBMs were ranked according to their adjusted HR, and mesenchymal and classical subtypes were shown to be more malignant than neural and proneural subtypes. **(C)** By dividing GBMs into two subgroups according to the molecular subtypes (proneural and neural vs. classical and mesenchymal), there was no significant difference in overall survival (*P=*0.078687). However, there was still a distinction in the survival curves between the more malignant subtypes (classical and mesenchymal) and the less malignant subtypes (proneural and neural). **(D)** The expression levels of *BICD1* were significantly higher in classical and mesenchymal subtypes than in neural and proneural subtypes (^***^). **(E)** The classical and mesenchymal subtypes had a significantly greater proportion of high *BICD1* expression than the neural and proneural subtypes (classical: 112/143, mesenchymal: 85/156, proneural: 47/138, neural: 17/87) (*P<*0.00001).

The molecular classification of GBMs was defined by TCGA. They used the 840 differentially expressed gene signature to classify GBMs into 4 clinically relevant subtypes (proneural, neural, classical, and mesenchymal) [34]. The proneural subtype has better prognosis than other subtypes of GBMs (Supplementary Figure 1A) (Supplementary Table 2). The expression levels of *BICD1* varied with the molecular subclassification of GBMs (Figure 3A). These subtypes of GBMs were ranked according to their adjusted HR, and mesenchymal and classical subtypes were more malignant than neural and proneural subtypes (Figure 3B). By dividing GBMs into two subgroups according to the molecular classification (proneural and neural vs. classical and mesenchymal), the difference in overall survival was not significant (*P=*0.078687) (Figure 3C). However, there was still a distinction in the survival curves between the more malignant subtypes (classical and mesenchymal) and the less malignant (proneural and neural) subtypes. The expression levels of *BICD1* were significantly higher in classical and mesenchymal subtypes than in neural and proneural subtypes (Figure 3D). The percentage of high *BICD1* expression was significantly greater in classical and mesenchymal subtypes than in neural and proneural subtypes (classical: 112/142, mesenchymal: 85/156, proneural: 47/138, neural: 17/87) (*P<*0.00001) (Figure 3E) (Table 4).

### Comparisons of *BICD1* and *MGMT* expression in predicting the overall survival of patients in various glioma datasets

**Figure 4:**
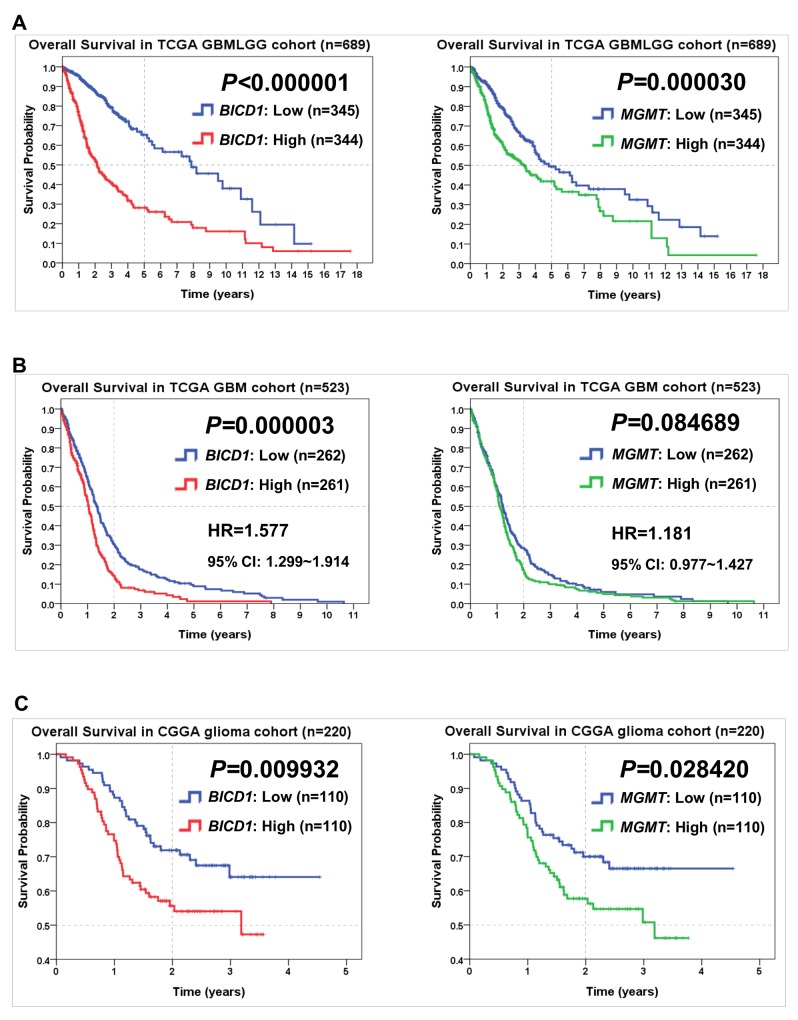
Comparisons of *BICD1* with *MGMT* expression in predicting the overall survival of patients in various glioma cohorts by the Kaplan-Meier survival analysis **(A)** High *BICD1* expression showed more significant impact on poor overall survival (*P<*0.000001) than high *MGMT* expression (*P=*0.000030) in the TCGA GBMLGG cohort. **(B)** High *BICD1* expression showed highly significant impact on poor overall survival (HR=1.577, *P=*0.000003), while high *MGMT* expression did not show significance (HR=1.181, *P=*0.084689) in the TCGA GBM cohort. **(C)** High *BICD1* expression showed more significant impact on poor overall survival (*P=*0.009932) than high *MGMT* expression (*P=*0.028420) in the CGGA glioma cohort.

In comparison of *BICD1* with *MGMT* expression in predicting the overall survival of glioma patients by the Kaplan-Meier survival analysis, high *BICD1* expression showed more significant impact (*P<*0.000001) than high *MGMT* expression (*P=*0.00003) on worse overall survival in the TCGA GBMLGG cohort (Figure 4A). High *BICD1* expression showed significant impact (*P=*0.000003), while high *MGMT* expression did not show significance (*P=*0.084689) on worse overall survival in the TCGA GBM cohort (Figure 4B). High *BICD1* expression also showed more significant impact (*P=*0.009932) than high *MGMT* expression (*P=*0.028420) on worse overall survival in the CGGA (Chinese Glioma Genome Atlas) cohort (Figure 4C).

### Comparisons of *BICD1* and *MGMT* expression in predicting the time to experience a new tumor event, the time to experience tumor progression, and the time to experience tumor recurrence in the TCGA GBM dataset

**Figure 5:**
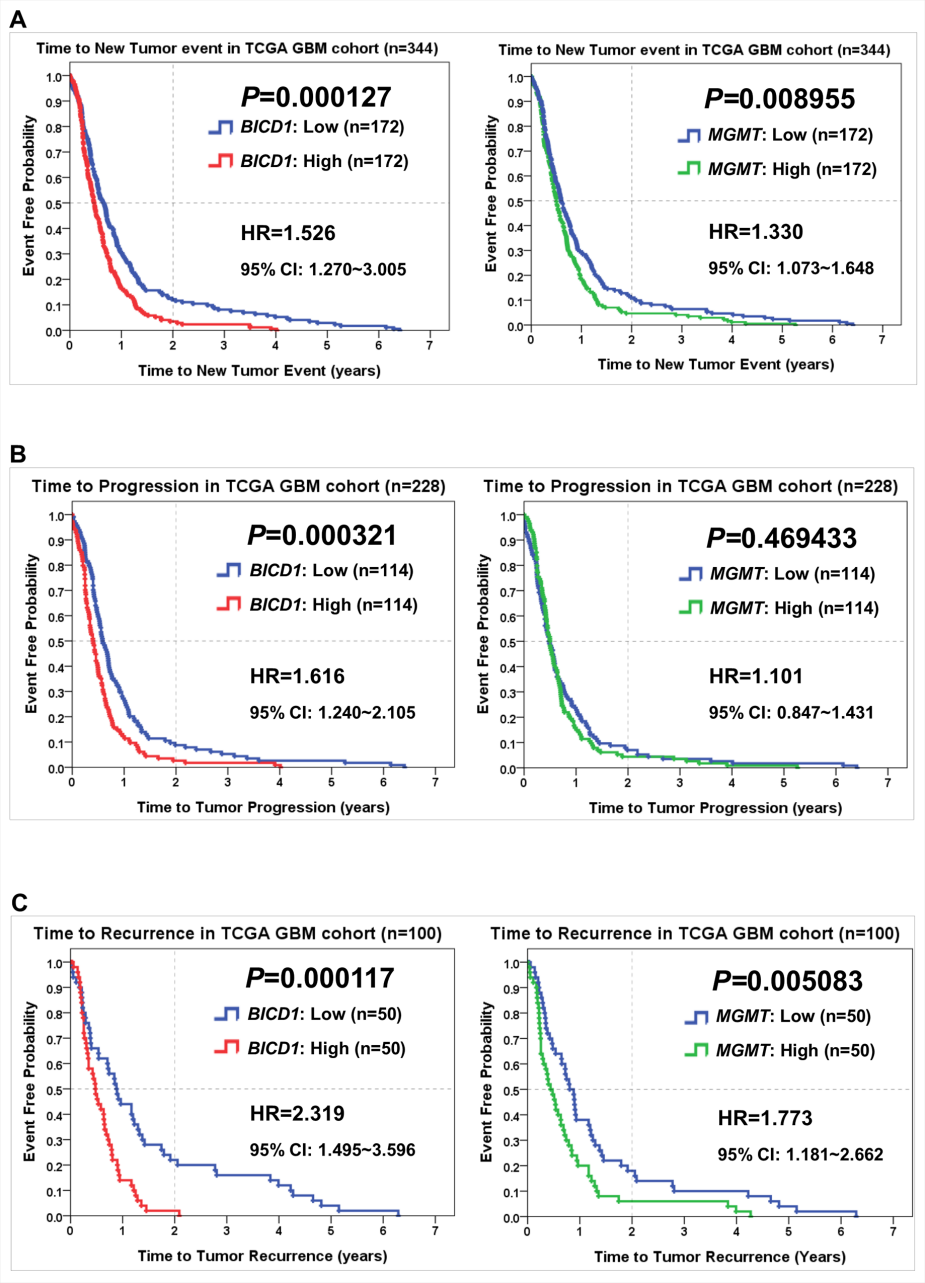
Comparisons of *BICD1* with *MGMT* expression in predicting other survival events of patients in the TCGA GBM cohort by the Kaplan-Meier survival analysis **(A)** GBM patients with high *BICD1* expression spent a significantly shorter time to experience a new tumor event (HR=1.526, *P=*0.000127) than those with high *MGMT* expression (HR=1.330, *P=*0.008955). **(B)** GBM patients with high *BICD1* expression spent a significantly shorter time to experience tumor progression (HR=1.616, *P=*0.000321), while those with high *MGMT* expression did not show significance (HR=1.101, *P=*0.469433). **(C)** GBM patients with high *BICD1* expression spent a significantly shorter time to experience tumor recurrence (HR=2.319, *P=*0.000117) than those with high *MGMT* expression (HR=1.773, *P=*0.005083).

Comparisons of *BICD1* with *MGMT* expression in predicting other survival events of patients in the TCGA GBM cohort were also made by the Kaplan-Meier survival analysis. The time to experience a new tumor event was significantly shorter in patients with high *BICD1* expression (*P=*0.000127) than in those with high *MGMT* expression (*P=*0.008955) (Figure 5A). The time to experience tumor progression was also significantly shorter in patients with high *BICD1* expression (*P=*0.000321), while it was not significantly shorter in patients with high *MGMT* expression (*P=*0.469433) (Figure 5B). And the time to experience tumor recurrence was significantly shorter in patients with high *BICD1* expression (*P=*0.000117) than in those with high *MGMT* expression (*P=*0.005083) (Figure 5C).

### Comparisons of *BICD1* and *MGMT* expression in predicting the response to various therapies in the TCGA GBM cohort

**Figure 6:**
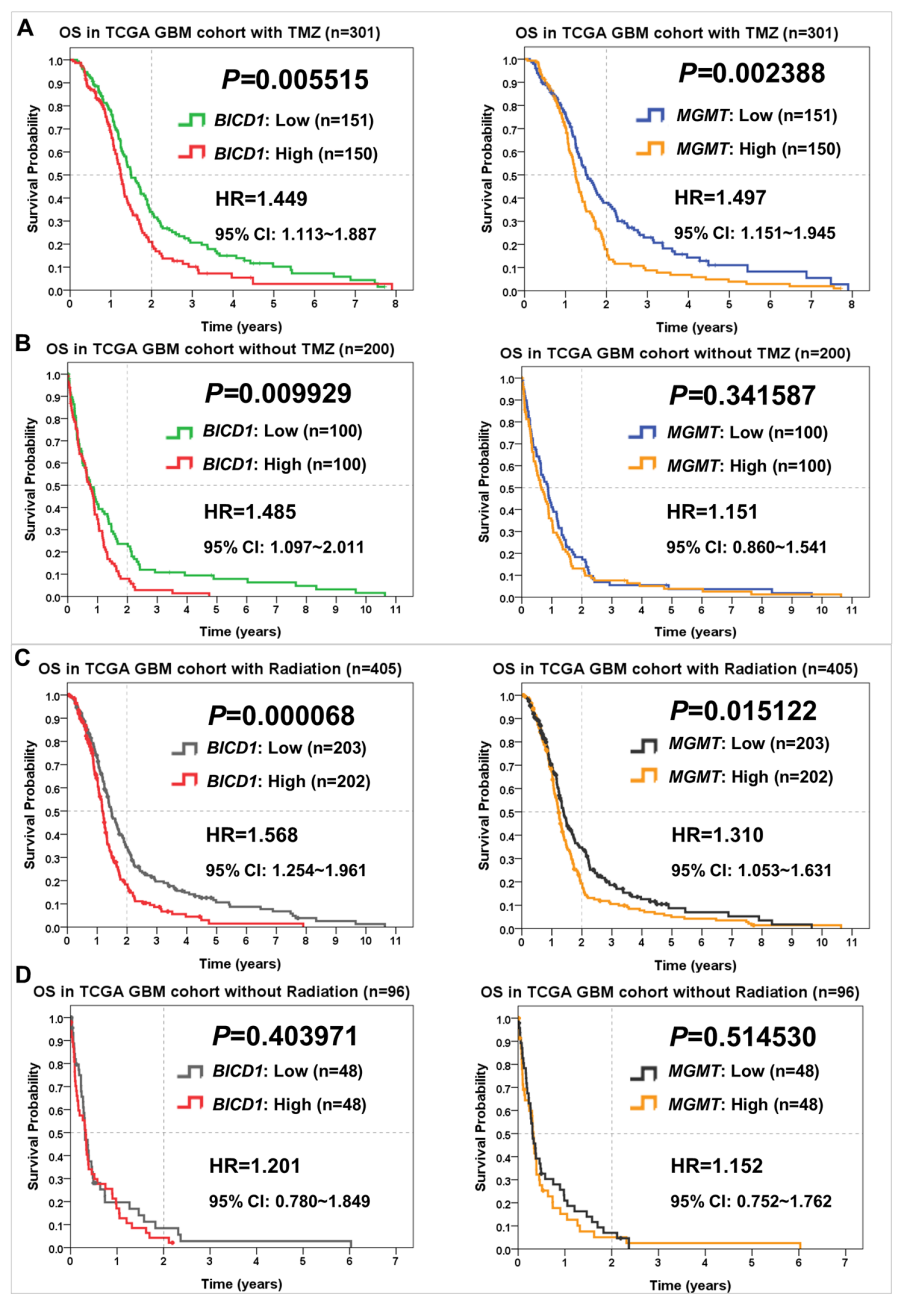
Comparisons of *BICD1* with *MGMT* expression in predicting the therapeutic outcomes of TMZ and radiation therapies in the TCGA GBM cohort by the Kaplan-Meier survival analysis (OS: overall survival) **(A)** In GBM patients who received TMZ chemotherapy, high *MGMT* expression showed more significant impact on poor overall survival (HR=1.497, *P=*0.002388) than high *BICD1* expression (HR=1.449, *P=*0.005515). **(B)** In GBM patients who did not receive TMZ chemotherapy, high *BICD1* expression still showed significant impact on poor overall survival (HR=1.485, *P=*0.009929), while high *MGMT* expression did not show significance (HR=1.151, *P=*0.341587). **(C)** In GBM patients who received radiation therapy, high *BICD1* expression showed more significant impact on poor overall survival (HR=1.568, *P=*0.000068) than high *MGMT* expression (HR=1.310, *P=*0.015122). **(D)** In GBM patients who did not receive radiation therapy, both *BICD1* and *MGMT* expression did not show significant impact on overall survival, but *BICD1* expression showed higher impact (HR=1.201, *P=*0.403971) than *MGMT* expression (HR=1.152, *P=*0.514530).

*BICD1* expression, as well as *MGMT,* were both powerful predictors of the response to TMZ treatment in GBM patients. In patients who received TMZ treatment, high *MGMT* expression showed significantly stronger impact (*P=*0.002388) on poor overall survival than high *BICD1* expression (*P=*0.005515) (Figure 6A). In patients without TMZ treatment, high *BICD1* expression still showed significant impact (*P=*0.009929) on poor overall survival, while high *MGMT* expression did not show significance (*P=*0.341587) (Figure 6B), which was compatible with the current knowledge that *MGMT* is a specific biomarker for predicting the response to TMZ treatment in glioma patients [35].

*BICD1* expression was a more powerful predictor of the response to radiation therapy in GBM patients than *MGMT* expression. In patients who received radiation therapy, high *BICD1* expression showed more significant impact (*P=*0.000068) on poor overall survival than high *MGMT* expression (*P=*0.015122) (Figure 6C). In patients without radiation therapy, both *BICD1* and *MGMT* expression did not show significant impact on the overall survival of GBM patients, but *BICD1* expression showed higher impact (*P=*0.403971) than *MGMT* expression (*P=*0.514530) (Figure 6D).

### Multivariate Cox regression analyses confirmed *BICD1* expression as an independent factor affecting the prognosis and response to therapies, including TMZ and radiation therapies in GBM patients

*BICD1* expression was an independent factor affecting the overall survival of GBM patients (adjusted HR=1.557, *P=*0.000009). Its impact power was more significant than *MGMT* expression (adjusted HR=1.291, *P=*0.008976), but less significant than age (adjusted HR=2.143, *P<*0.000001) (Table 5).

**Table 5:** Univariate and multivariate Cox regression analyses of *BICD1* expression and the clinicopathological factors in the overall survival of patients in the TCGA GBM cohort

Variables		Univariate			Multivariate		
		HR	95% CI	*P*	HR	95% CI	*P*
***BICD1***	Low vs. High	1.577	1.299-1.914	**0.000004**	1.557	1.281-1.894	**0.000009**
**Age**	<65 vs. ≥65	2.139	1.741-2.627	**<0.000001**	2.143	1.742-2.638	**<0.000001**
**Gender**	Female vs. Male	1.175	0.967-1.429	0.104814	1.113	0.915-1.353	0.284136
***MGMT***	Low vs. High	1.181	0.977-1.427	0.085370	1.291	1.066-1.563	**0.008976**

HR: hazard ratio, CI: confidence interval.

*BICD1* expression was an independent factor affecting the response to TMZ treatment in GBM patients (adjusted HR=1.576, *P=*0.000974). However, age (adjusted HR=1.777, *P=*0.000211) and *MGMT* expression (adjusted HR=1.647, *P=*0.000270) showed more significance than *BICD1* expression in affecting the response to TMZ treatment (Table 6).

**Table 6:** Univariate and multivariate Cox regression analyses of *BICD1* expression and the clinicopathological factors in the overall survival of patients with TMZ treatment in the TCGA GBM cohort

Variables		Univariate			Multivariate		
		HR	95% CI	*P*	HR	95% CI	*P*
***BICD1***	Low vs. High	1.449	1.113-1.887	**0.005826**	1.576	1.203-2.065	**0.000974**
**Age**	<65 vs. ≥65	1.746	1.290-2.364	**0.000309**	1.777	1.311-2.408	**0.000211**
**Gender**	Female vs. Male	1.247	0.949-1.639	0.113591	1.235	0.938-1.626	0.133230
***MGMT***	Low vs. High	1.497	1.151-1.945	**0.002575**	1.647	1.259-2.154	**0.000270**

HR: hazard ratio, CI: confidence interval.

*BICD1* expression was also an independent factor affecting the overall survival of GBM patients who did not receive TMZ treatment (adjusted HR=1.416, *P=*0.027711). Its impact power was more significant than *MGMT* expression (adjusted HR=1.110, *P*=0.487235), but less significant than age (adjusted HR=2.250, *P<*0.000001) (Table 7).

**Table 7:** Univariate and multivariate Cox regression analyses of *BICD1* expression and the clinicopathological factors in the overall survival of patients without TMZ treatment in the TCGA GBM cohort

Variables		Univariate			Multivariate		
		HR	95% CI	*P*	HR	95% CI	*P*
***BICD1***	Low vs. High	1.485	1.097-2.011	**0.010485**	1.416	1.039-1.929	**0.027711**
**Age**	<65 vs. ≥65	2.286	1.682-3.107	**<0.000001**	2.250	1.655-3.059	**<0.000001**
**Gender**	Female vs. Male	1.181	0.878-1.589	0.271452	1.101	0.815-1.487	0.532466
***MGMT***	Low vs. High	1.151	0.860-1.541	0.342714	1.110	0.827-1.488	0.487235

HR: hazard ratio, CI: confidence interval.

*BICD1* expression was an independent factor affecting the response to radiation therapy in GBM patients (adjusted HR=1.601, *P=*0.000044). Age (adjusted HR=1.676, *P=*0.000072) and *MGMT* expression (adjusted HR=1.414, *P=*0.002191) were also significant factors, but not as significant as *BICD1* expression (Table 8).

**Table 8:** Univariate and multivariate Cox regression analyses of *BICD1* expression and the clinicopathological factors in the overall survival of patients with radiation therapy in the TCGA GBM cohort

Variables		Univariate			Multivariate		
		HR	95% CI	*P*	HR	95% CI	*P*
***BICD1***	Low vs. High	1.568	1.254-1.961	**0.000078**	1.601	1.277-2.007	**0.000044**
**Age**	<65 vs. ≥65	1.657	1.287-2.134	**0.000091**	1.676	1.299-2.162	**0.000072**
**Gender**	Female vs. Male	1.215	0.968-1.524	0.093019	1.138	0.906-1.430	0.265744
***MGMT***	Low vs. High	1.310	1.053-1.631	**0.015538**	1.414	1.133-1.765	**0.002191**

HR: hazard ratio, CI: confidence interval.

*BICD1* (adjusted HR=1.280, *P=*0.267624) and *MGMT* expression (adjusted HR=1.115, *P=*0.620605) were not independent factors affecting the overall survival of GBM patients who did not receive radiation therapy, while age was still a significant factor (adjusted HR=2.126, *P=*0.001580), which suggested that *BICD1* and *MGMT* expression may be specific biomarkers for predicting the response to radiation therapy (Table 9).

**Table 9:** Univariate and multivariate Cox regression analyses of *BICD1* expression and the clinicopathological factors in the overall survival of patients without radiation therapy in the TCGA GBM cohort

Variables		Univariate			Multivariate		
		HR	95% CI	*P*	HR	95% CI	*P*
***BICD1***	Low vs. High	1.201	0.780-1.849	0.405628	1.280	0.827-1.980	0.267624
**Age**	<65 vs. ≥65	2.044	1.283-3.256	**0.001638**	2.126	1.332-3.395	**0.001580**
**Gender**	Female vs. Male	1.274	0.825-1.969	0.274642	1.363	0.879-2.112	0.166421
***MGMT***	Low vs. High	1.152	0.752-1.762	0.515767	1.115	0.725-1.715	0.620605

HR: hazard ratio, CI: confidence interval.

### Risk stratification of GBM patients according to the combination of age and *BICD1* expression may provide more accurate outcome assessment

**Figure 7:**
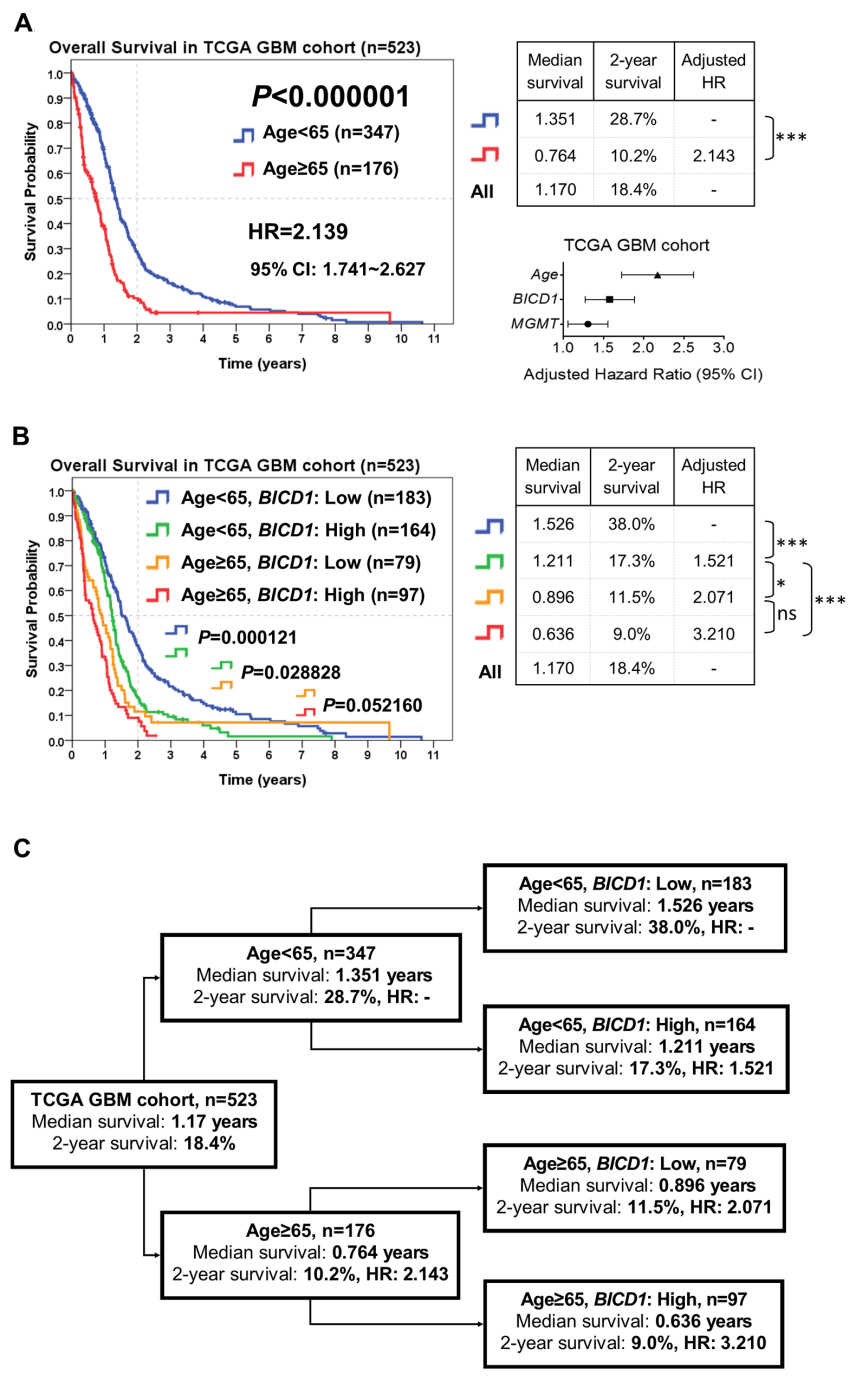
The combined utilization of age and *BICD1* expression may provide more accurate outcome assessment of GBM patients by risk stratification of patients in the TCGA GBM cohort (n=523) The median survival, 2-year survival rate and adjusted HR were obtained by the Kaplan-Meier survival analysis. **(A)** Age was highly significant in determining the outcomes of GBM patients (*P<*0.000001). Patients with higher age (≥65) had poorer outcomes (adjusted HR=2.143) than those with lower age (<65). The difference in the overall survival of patients between age<65 and ≥65 was highly significant. The median survival and 2-year survival rate in patients with age<65 were 1.351 years and 28.7%, and in patients with age≥65 were 0.764 years and 10.2%, respectively. In addition to age, *BICD1* expression was found to have a high adjusted HR (adjusted HR=1.557) in GBM patients (Table 5). **(B)** By adding *BICD1* expression as a cofactor with age, patients with age<65 could be further divided into higher and lower risk groups (median survival: 1.211 vs. 1.526 years, 2-year survival rate: 17.3% vs. 38.0%) (*P=*0.000121). Patients with age≥65 could also be divided into higher and lower risk groups (median survival: 0.636 vs. 0.896 years, 2-year survival rate: 9.0% vs. 11.5%) (*P=*0.052160). The patient group with age≥65 and high *BICD1* expression had the highest HR and the poorest prognosis (adjusted HR=3.210, median survival: 0.636 years, and 2-year survival rate: 9.0%). **(C)** A flowchart to represent the application of our results in Figure 7B. This may provide more accurate prediction of patients’ median survival, 2-year survival rate, and adjusted HR when age and *BICD1* expression were combinedly utilized for risk stratification of GBM patients.

According to the combination of patient age with *BICD1* expression, patients in the TCGA GBM cohort (n=523) were stratified into more distinct risk groups for outcome assessment by the Kaplan-Meier survival analysis. The median survival, 2-year survival rate, and HR of GBM patients in each subgroup were listed. The difference in the overall survival of patients between age<65 and ≥65 was highly significant (*P<*0.000001). Patients with higher age (≥65) had poorer outcomes (adjusted HR=2.143) than those with lower age (<65) (Figure 7A). The median survival and 2-year survival rate in patients with age<65 were 1.351 years and 28.7%, and in patients with age≥65 were 0.764 years and 10.2%, respectively. In addition to age, *BICD1* expression was shown to have a high HR (adjusted HR=1.557) in patients with GBMs (Table 5).

By adding *BICD1* expression as a cofactor with age, patients with age<65 could be further divided into higher and lower risk groups (median survival: 1.211 vs. 1.526 years, 2-year survival rate: 17.3% vs. 38.0%) (*P=*0.000121). Patients with age≥65 could also be divided into higher and lower risk groups (median survival: 0.636 vs. 0.896 years, 2-year survival rate: 9.0% vs. 11.5%) (*P=*0.052160) (Figure 7B). The patient group with age≥65 and high *BICD1* expression had the highest HR and the poorest prognosis (adjusted HR=3.210, median survival: 0.636 years, and 2-year survival rate: 9.0%). And the patient group with age<65 and low *BICD1* expression had the best prognosis (median survival: 1.526 years, and 2-year survival rate: 38.0%).

The flowchart in Figure 7C represents the application of our results in Figure 7B. This may provide more accurate prediction of patients’ prognosis when age and *BICD1* expression were combinedly utilized for risk stratification of GBM patient (Figure 7C).

### Risk stratification of GBM patients who received TMZ chemotherapy according to the combination of age, *MGMT,* and *BICD1* expression may provide better prediction of the response to TMZ

**Figure 8:**
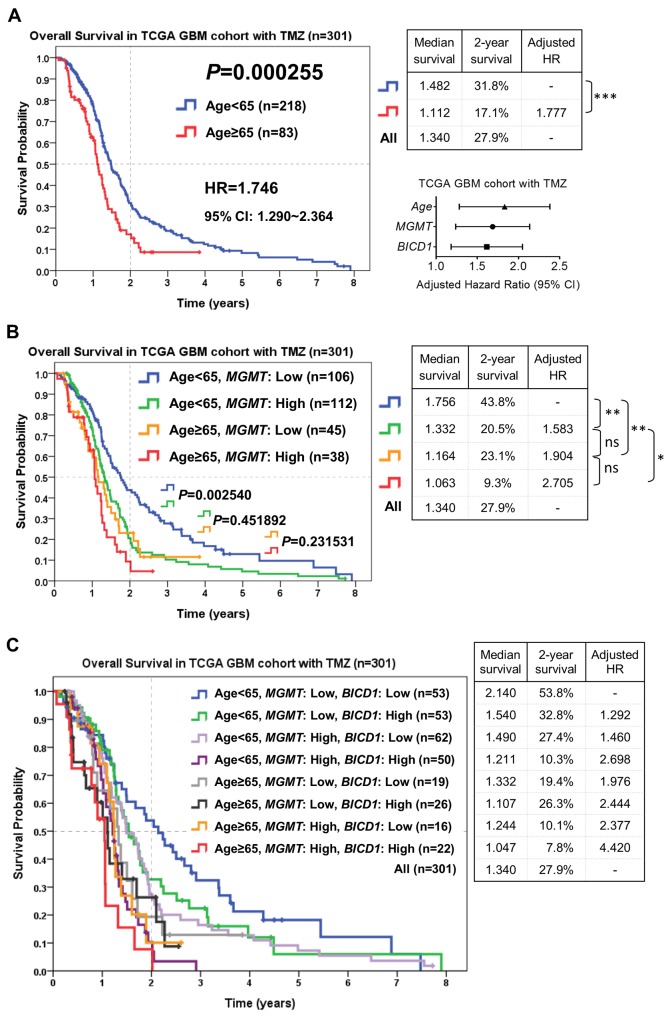
The combined utilization of age, *MGMT* and *BICD1* expression may provide better prediction of patients’ response to TMZ chemotherapy by risk stratification of GBM patients who received TMZ chemotherapy in the TCGA GBM cohort (n=301) The median survival, 2-year survival rate and adjusted HR were obtained by the Kaplan-Meier survival analysis. **(A)** Age was highly significant in determining the outcomes of patients who received TMZ chemotherapy (*P=*0.000255). Patients with higher age (≥65) had poorer outcomes (adjusted HR=1.777) than those with lower age (<65). In addition to age, *MGMT* and *BICD1* expression were found to have high adjusted HRs (*MGMT*: adjusted HR=1.647, *BICD1*: adjusted HR=1.576) in GBM patients with TMZ treatment (Table 6). **(B)** The difference in the overall survival of patients with age<65 between low and high *MGMT* expression was significant (*P=*0.002540). The patient group with age<65 and low *MGMT* expression had the best prognosis (median survival: 1.756 years, and 2-year survival rate: 43.8%). The patient group with age≥65 and high *MGMT* expression had the highest HR and the poorest prognosis (adjusted HR=2.705, median survival: 1.063 years, and 2-year survival rate: 9.3%). **(C)** By adding *BICD1* expression as a cofactor with age and *MGMT* expression, the patient group with age<65, low *MGMT* and low *BICD1* expression had the best prognosis (median survival: 2.140 years, and 2-year survival rate: 53.8%). The patient group with age≥65, high *MGMT* and high *BICD1* expression had the highest HR and the poorest prognosis (adjusted HR=4.420, median survival: 1.047 years, and 2-year survival rate: 7.8%).

According to the combination of age, *MGMT*, and *BICD1* expression, patients who received TMZ chemotherapy in the TCGA GBM cohort (n=301) were stratified into distinct risk groups for outcome assessment by the Kaplan-Meier survival analysis. The median survival, 2-year survival rate, and HR of patients in each subgroup were listed. Age was highly significant in determining the outcomes of GBM patients who received TMZ chemotherapy (*P=*0.000255). Patients with higher age (age≥65) had poorer outcomes (adjusted HR=1.777) than those with lower age (<65) (Figure 8A). The median survival and 2-year survival rate in patients with age<65 were 1.482 years and 31.8%, and in patients with age≥65 were 1.112 years and 17.1%, respectively. In addition to age, *MGMT* and *BICD1* expression were shown to have high HRs (*MGMT*: adjusted HR=1.647, *BICD1*: adjusted HR=1.576) in GBM patients with TMZ treatment (Table 6).

By adding *MGMT* expression as a cofactor with age, patients with age<65 could be further divided into higher and lower risk groups (median survival: 1.332 vs. 1.756 years, 2-year survival rate: 20.5% vs. 43.8%) (*P=*0.002540). Patients with age≥65 could also be divided into higher and lower risk groups (median survival: 1.063 vs. 1.164 years, 2-year survival rate: 9.3% vs. 23.1%) (*P=*0.231531) (Figure 8B). The patient group with age≥65 and high *MGMT* expression had the highest HR and the poorest prognosis (adjusted HR=2.705, median survival: 1.063 years, and 2-year survival rate: 9.3%). And the patient group with age<65 and low *MGMT* expression had the best prognosis (median survival: 1.756 years, and 2-year survival rate: 43.8%).

By adding *BICD1* expression as a cofactor with age and *MGMT* expression, the patient group with age<65 and low *MGMT* expression could be further divided into higher and lower risk groups (median survival: 1.540 vs. 2.140 years, 2-year survival rate: 32.8% vs. 53.8%), the patient group with age<65 and high *MGMT* expression could be further divided into higher and lower risk groups (median survival: 1.211 vs. 1.490 years, 2-year survival rate: 10.3% vs. 27.4%), the patient group with age≥65 and low *MGMT* expression could be further divided into higher and lower risk groups (median survival: 1.107 vs. 1.332 years, 2-year survival rate: 26.3% vs. 19.4%), and the patient group with age≥65 and high *MGMT* expression could be further divided into higher and lower risk groups (median survival: 1.047 vs. 1.244 years, 2-year survival rate: 7.8% vs. 10.1%) (Figure 8C). The patient group with age≥65, high *MGMT* and high *BICD1* expression had the highest HR and the poorest prognosis (adjusted HR=4.420, median survival: 1.047 years, and 2-year survival rate: 7.8%). The patient group with age<65, low *MGMT* and low *BICD1* expression had the best prognosis (median survival: 2.140 years, and 2-year survival rate: 53.8%).

**Figure 9:**
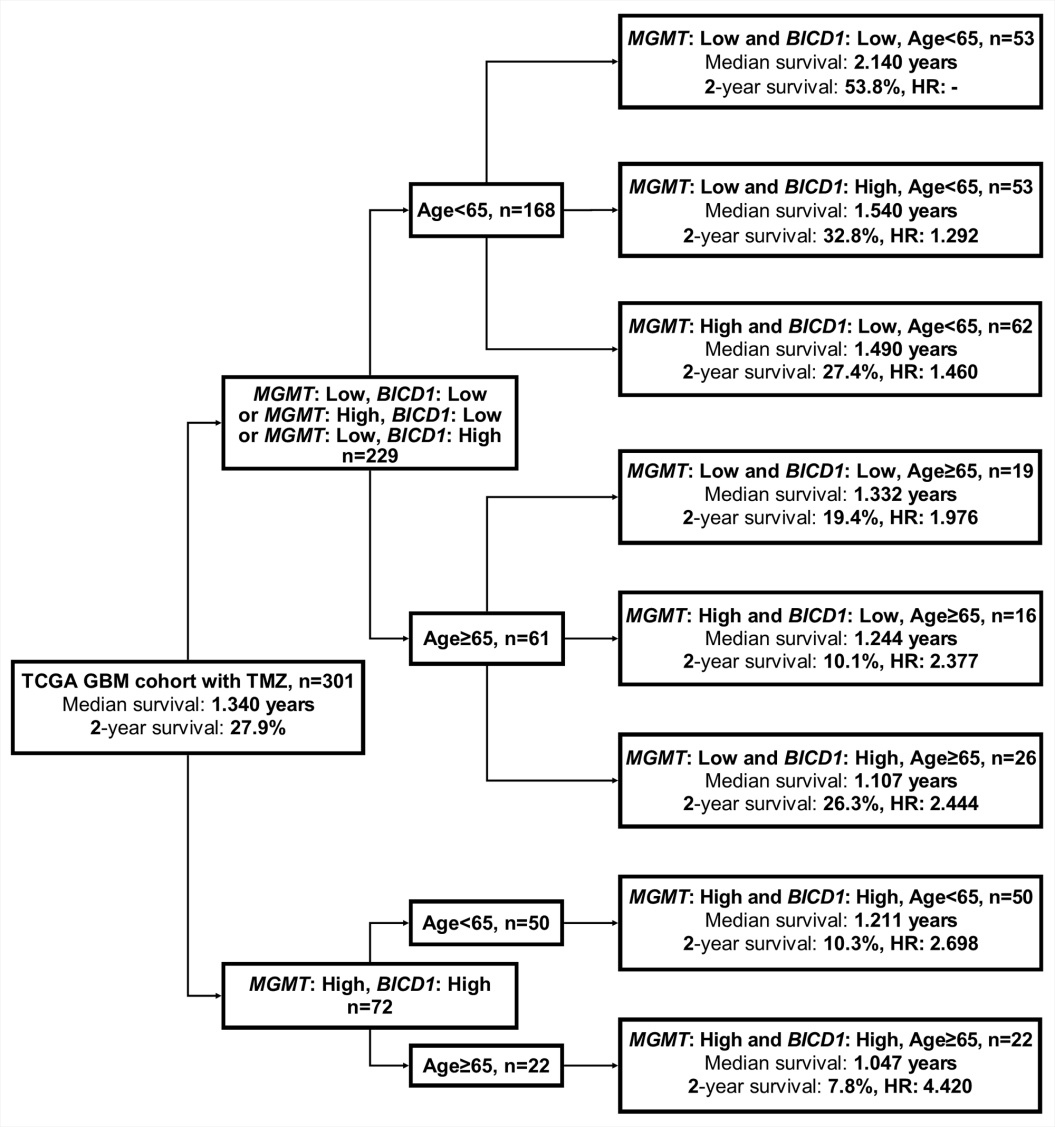
A flowchart to represent the application of our results in Figure 8C This may provide better prediction of patients’ median survival, 2-year survival rate, and adjusted HR when age, *MGMT* and *BICD1* expression were combinedly utilized for risk stratification of GBM patients who received TMZ chemotherapy. First, GBM patients who received TMZ chemotherapy were separated into 2 subgroups according to whether they had low expression of either *BICD1* or *MGMT* or both, and high expression of both *MGMT* and *BICD1*. The patient group with high expression of both genes could be further stratified into 2 distinct risk groups according to age. The patient group with low expression of either *BICD1* or *MGMT* or both, were further stratified into 2 subgroups according to age, and in each age group (age<65 and age≥65), patients were further stratified into 3 distinct risk groups according to their *MGMT* and *BICD1* expression status.

The flowchart in Figure 9 represents the application of our results in Figure 8C. This may provide better prediction of GBM patients’ response to TMZ chemotherapy when age, *MGMT* and *BICD1* expression were combinedly utilized for risk stratification of GBM patients who received TMZ chemotherapy. First, GBM patients with TMZ chemotherapy were separated according to whether they had low expression of either *BICD1* or *MGMT* or both, and high expression of both *MGMT* and *BICD1*. Second, the patient group with high expression of both *MGMT* and *BICD1* could be further stratified into 2 distinct risk groups according to age. Third, the patient group with low expression of either *BICD1* or *MGMT* or both were also stratified into 2 distinct subgroups according to age, and in each age group (age<65 or age≥65), patients were further stratified into 3 distinct risk groups according to *MGMT* and *BICD1* expression (Figure 9).

### Risk stratification of GBM patients who received radiation therapy according to the combination of age and *BICD1* expression may provide better prediction of the response to radiation

**Figure 10:**
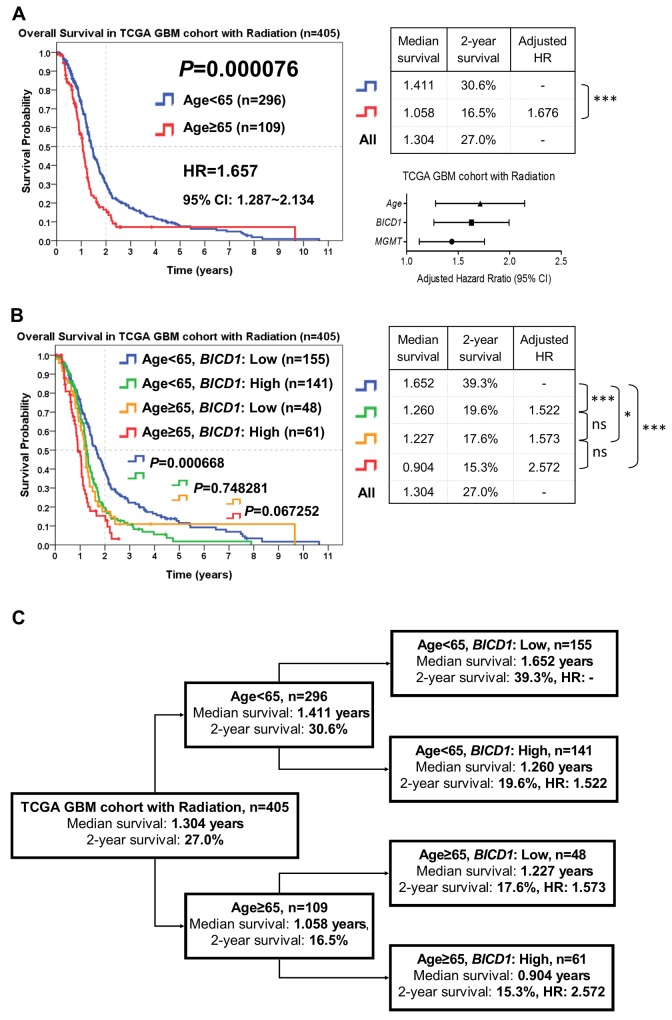
The combined utilization of age and *BICD1* expression may provide better prediction of patients’ response to radiation therapy by risk stratification of GBM patients who received radiation therapy in the TCGA GBM cohort (n=405) The median survival, 2-year survival rate, and adjusted HR were obtained by the Kaplan-Meier survival analysis. **(A)** Age was highly significant in determining the outcomes of patients who received radiation therapy (*P=*0.000076). Patients with higher age (≥65) had poorer outcomes (adjusted HR=1.676) than those with lower age (<65). In addition to age, *BICD1* expression was found to have a high adjusted HR (adjusted HR=1.601) in GBM patients with radiation therapy (Table 8). **(B)** The difference in the overall survival of patients with age<65 between low and high *BICD1* expression was highly significant (*P=*0.000668). By adding *BICD1* expression as a cofactor with age, patients with age<65 could be divided into higher and lower risk groups (median survival: 1.260 vs. 1.652 years, 2-year survival rate: 19.6% vs. 39.3%) (*P=*0.000668). Patients with age≥65 could also be divided into higher and lower risk groups (median survival: 0.904 vs. 1.227 years, 2-year survival rate: 15.3% to 17.6%) (*P=*0.067252). The patient group with age≥65 and high *BICD1* expression had the highest HR and the poorest prognosis (adjusted HR=2.572, median survival: 0.904 years, and 2-year survival rate: 15.3%). And the patient group with age<65 and low *BICD1* expression had the best prognosis (median survival: 1.652 years, and 2-year survival rate: 39.3%). **(C)** A flowchart to represent the application of our results in Figure 10B. This may provide better prediction of patients’ median survival, 2-year survival rate, and adjusted HR when age and *BICD1* expression were combinedly utilized for risk stratification of GBM patients who received radiation therapy.

According to the combination of age with *BICD1* expression, patients who received radiation therapy in the TCGA GBM cohort (n=405) were stratified into distinct risk groups for outcome assessment by the Kaplan-Meier survival analysis. Age was highly significant in determining the outcomes of GBM patients who received radiation therapy (*P=*0.000076), and patients with higher age (≥65) had poorer outcomes (adjusted HR=1.676) than those with lower age (<65) (Figure 10A). The median survival and 2-year survival rate in patients with age<65 were 1.411 years and 30.6%, and in patients with age≥65 were 1.058 years and 16.5%, respectively. In addition to age, *BICD1* expression was found to have a high HR (adjusted HR=1.601) in GBM patients with radiation therapy (Table 8).

By adding *BICD1* expression as a cofactor with age, patients with age<65 could be divided into higher and lower risk groups (median survival: 1.260 vs. 1.652 years, 2-year survival rate: 19.6% vs. 39.3%) (*P=*0.000668). Patients with age≥65 could also be divided into higher and lower risk groups (median survival: 0.904 vs. 1.227 years, 2-year survival rate: 15.3% to 17.6%) (*P=*0.067252) (Figure 10B). The patient group with age≥65 and high *BICD1* expression had the highest HR and the poorest prognosis (adjusted HR=2.572, median survival: 0.904 years, and 2-year survival rate: 15.3%). And the patient group with age<65 and low *BICD1* expression had the best prognosis (median survival: 1.652 years, and 2-year survival rate: 39.3%).

The flowchart in Figure 10C represents the application of our results in Figure 10B. This may provide better prediction of GBM patients’ response to radiation therapy when age and *BICD1* expression were combinedly utilized for risk stratification of GBM patients who received radiation therapy (Figure 10C).

### The possible mechanisms of *BICD1*-associated survival or therapeutic resistance in GBM cells

**Figure 11:**
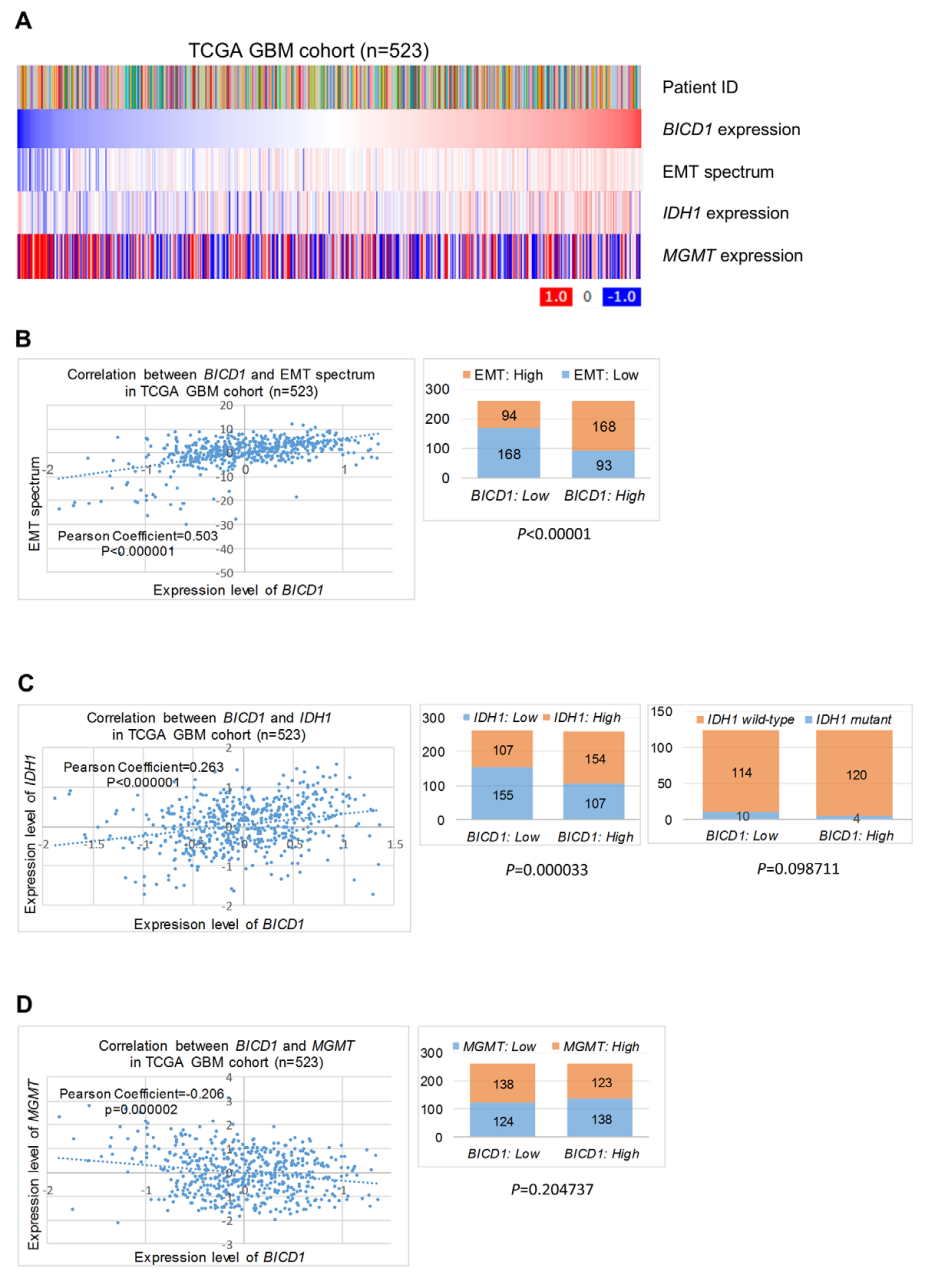
The possible mechanisms of *BICD1*-associated survival or therapeutic resistance in GBMs **(A)** *BICD1* expression was positively correlated with the EMT spectrum and *IDH1* expression, but appeared to be negatively correlated with *MGMT* expression in the TCGA GBM cohort (n=523). **(B)** The expression levels of *BICD1* were positively and significantly correlated with the EMT spectrum (*P<*0.000001, Pearson’s correlation coefficient=0.503). Patients with high *BICD1* expression had a significantly greater proportion of high EMT spectrum than those with low *BICD1* expression (High *BICD1* expression: 168/261 vs. Low *BICD1* expression: 94/262) (*P<*0.00001). **(C)** The expression levels of *BICD1* and *IDH1* were positively and significantly correlated in the TCGA GBM cohort (*P<*0.000001, Pearson’s correlation coefficient=0.263). Patients with high *BICD1* expression had a significantly greater proportion of high *IDH1* expression than those with low *BICD1* expression (High *BICD1* expression: 154/261 vs. Low *BICD1* expression: 107/262) (*P=*0.000033). Patients with high *BICD1* expression had a greater proportion of *IDH1* wild-type than those with low *BICD1* expression, which was not statistically significant (High *BICD1* expression: 120/124 vs. Low *BICD1* expression: 114/124) (*P=*0.098711). **(D)** The expression levels of *BICD1* and *MGMT* were significantly and negatively correlated in the TCGA GBM cohort (*P=*0.000002, Pearson’s correlation coefficient=-0.206). Patients with high *BICD1* expression had a smaller proportion of high *MGMT* expression than those with low *BICD1* expression, which was not statistically significant (High *BICD1* expression: 123/261 vs. Low *BICD1* expression: 138/262) (*P=*0.204737).

The possible mechanisms of *BICD1*-associated survival or therapeutic resistance in GBM cells were proposed according to our observations. *BICD1* expression was positively correlated with the EMT spectrum and *IDH1* expression, but appeared to be negatively correlated with *MGMT* expression in the TCGA GBM cohort (n=523) (Figure 11A). The expression levels of *BICD1* were significantly and positively correlated with the EMT spectrum in the TCGA GBM cohort (*P<*0.000001, Pearson’s correlation coefficient=0.503). Patients with high *BICD1* expression had a significantly higher percentage of high EMT spectrum than those with low *BICD1* expression (High *BICD1* expression: 168/261 vs. Low *BICD1* expression: 94/262) (*P<*0.00001) (Figure 11B). The expression levels of *BICD1* and *IDH1* were also significantly and positively correlated in the TCGA GBM cohort (*P<*0.000001, Pearson’s correlation coefficient=0.263). Patients with high *BICD1* expression had a significantly higher percentage of high *IDH1* expression than those with low *BICD1* expression (High *BICD1* expression: 154/261 vs. Low *BICD1* expression: 107/262) (*P=*0.000033). Patients with high *BICD1* expression had a higher percentage of *IDH1* wild-type than those with low *BICD1* expression, however, which was not statistically significant (High *BICD1* expression: 120/124 vs. Low *BICD1* expression: 114/124) (*P=*0.098711) (Figure 11C). The expression levels of *BICD1* and *MGMT* were significantly but negatively correlated in the TCGA GBM cohort (*P=*0.000002, Pearson’s correlation coefficient=-0.206). Patients with high *BICD1* expression had a lower percentage of high *MGMT* expression than those with low *BICD1* expression, however, which was not statistically significant (High *BICD1* expression: 123/261 vs. Low *BICD1* expression: 138/262) (*P=*0.204737) (Figure 11D). Our results suggested a significant correlation between *BICD1* expression and EMT.

**Figure 12:**
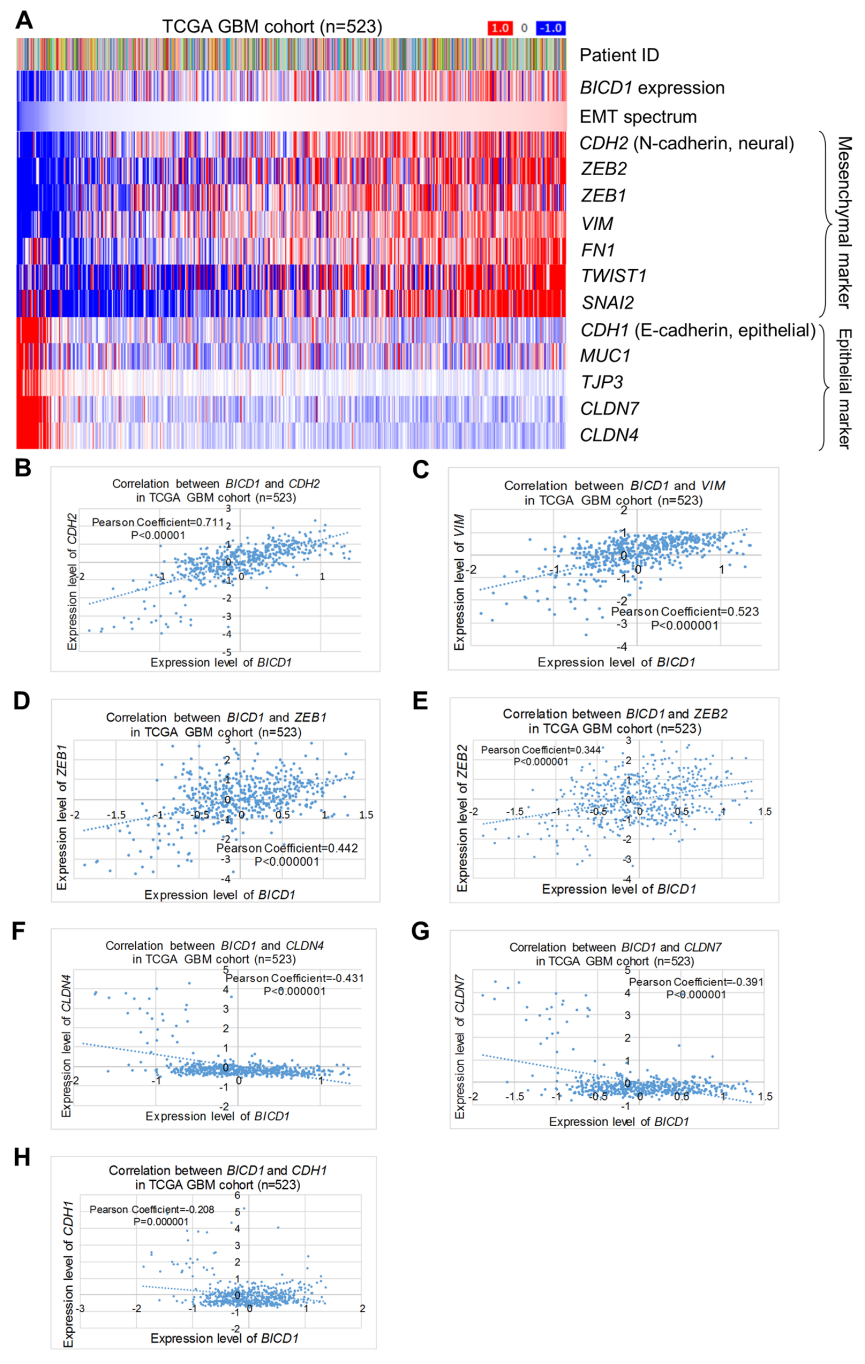
Correlations of *BICD1* with EMT-related markers in the TCGA GBM cohort (n=523) **(A)** *BICD1* expression was positively correlated with the EMT spectrum and expression of the mesenchymal markers of *CDH2, ZEB2, ZEB1, VIM, FN1, TWIST1,* and *SNAI2*, but negatively correlated with expression of the epithelial markers of *CDH1, MUC1, TJP3, CLDN7* and *CLDN4*. **(B)** The expression levels of *BICD1* were significantly and positively correlated with *CDH2* expression (*P<*0.000001, Pearson’s correlation coefficient=0.711). **(C)** The expression levels of *BICD1* were significantly and positively correlated with *VIM* expression (*P<*0.000001, Pearson’s correlation coefficient=0.523). **(D)** The expression levels of *BICD1* were significantly and positively correlated with *ZEB2* expression (*P<*0.000001, Pearson’s correlation coefficient=0.344). **(E)** The expression levels of *BICD1* were significantly and positively correlated with *ZEB1* expression (*P<*0.000001, Pearson’s correlation coefficient=0.442). **(F)** The expression levels of *BICD1* were significantly and negatively correlated with *CLDN7* expression (*P<*0.000001, Pearson’s correlation coefficient=-0.391). **(G)** The expression levels of *BICD1* were significantly and negatively correlated with *CLDN4* expression (*P<*0.000001, Pearson’s correlation coefficient=-0.431). **(H)** The expression levels of *BICD1* were significantly and negatively correlated with *CDH1* expression (*P=*0.000001, Pearson’s correlation coefficient=-0.208).

The correlations between expression of *BICD1* and EMT markers in the TCGA GBM cohort (n=523) were further analyzed. *BICD1* expression was positively correlated with the EMT spectrum and expression of the mesenchymal markers of *CDH2, ZEB2, ZEB1*, *VIM, FN1, TWIST1* and *SNAI2*, but negatively correlated with expression of the epithelial markers of *CDH1, MUC1, TJP3, CLDN7* and *CLDN4* (Figure 12A). The expression levels of *BICD1* were significantly and positively correlated with *CDH2* expression (*P<*0.000001, Pearson’s correlation coefficient=0.711) (Figure 12B), *VIM* expression (*P<*0.000001, Pearson’s correlation coefficient=0.523) (Figure 12C), *ZEB1* expression (*P<*0.000001, Pearson’s correlation coefficient=0.442) (Figure 12D), and *ZEB2* expression (*P<*0.000001, Pearson’s correlation coefficient=0.344) (Figure 12E), but negatively correlated with *CLDN4* expression (*P<*0.000001, Pearson’s correlation coefficient=-0.431) (Figure 12F), *CLDN7* expression (*P<*0.000001, Pearson’s correlation coefficient=-0.391) (Figure 12G), and *CDH1* expression (*P=*0.000001, Pearson’s correlation coefficient=-0.208) (Figure 12H). These results further proved the strong correlation between *BICD1* expression and EMT.

**Figure 13:**
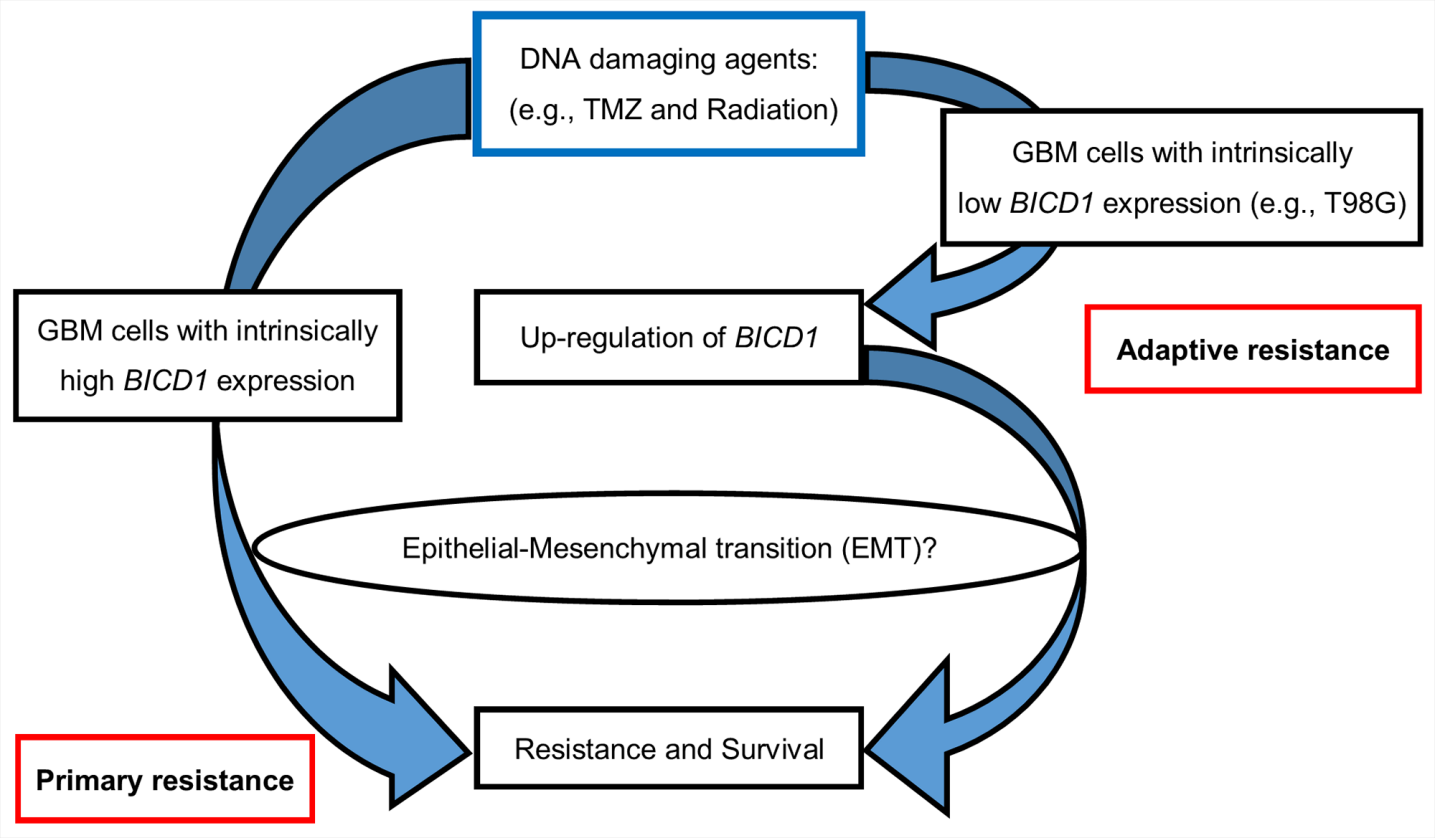
A proposed mechanism of *BICD1*-associated survival or therapeutic resistance in response to DNA-damaging agents (e.g., TMZ and radiation) in GBM cells On the left, the GBM cells with intrinsically high *BICD1* expression may have primary resistance to DNA damage through EMT. On the right, the GBM cells with originally low *BICD1* expression, after exposure to a DNA-damaging agent, may experience up-regulation of *BICD1,* and thus, acquire adaptive resistance to DNA damage via EMT.

The diagram in Figure 13 illustrated a proposed mechanism of *BICD1*-associated survival or therapeutic resistance in GBM cells when treated with DNA-damaging agents (e.g., TMZ and radiation). On the left, the GBM cells with intrinsically high *BICD1* expression may have primary resistance to DNA damage through EMT. On the right, the GBM cells with originally low *BICD1* expression, after exposure to a DNA-damaging agent, may experience up-regulation of *BICD1* and thus, acquire adaptive resistance to DNA damage via EMT.

## DISCUSSION

This is the first study to identify TMZ-related genomic markers using a cell-based microarray model. In our study, we identified *BICD1* gene expression as a TMZ-related genomic marker, which also showed high significance in prognosis and the response to therapies, including TMZ and radiation therapies, in GBM patients. In our results, high *BICD1* expression was significantly correlated with poor overall survival in GBM patients. Patients with high *BICD1* expression spent a significantly shorter time to experience a new tumor event, tumor progress, and tumor recurrence than those with low *BICD1* expression. The most impressive finding in this study is that *BICD1,* as well as *MGMT* expression, both showed high significance in predicting the outcomes of GBM patients with TMZ or radiation therapies. Finally, we confirmed *BICD1* expression as an independent factor affecting the prognosis and therapeutic response of TMZ and radiation in GBM patients. According to the combination of patient age, *MGMT*, and *BICD1* expression, GBM patients who received TMZ treatment could be further separated into more distinct risk groups, which may provide more predictive information of patients’ outcomes, and help neuro-oncologists make better individualized therapeutic decisions, and develop personalized therapy or precision medicine for GBM patients in the future.

Notably, in our microarray result, the most differentially expressed gene in T98G after TMZ treatment was the Far Upstream Element-Binding Protein 1 (*FUBP1)*, which suggested that *FUBP1* may play a certain role in response to TMZ treatment in GBM cells. *FUBP1* has been reported as a biomarker for nasopharyngeal carcinoma gliomas [36] and clear cell renal cell carcinoma [37], and previously described as a transcriptional regulator of the important proto-oncogene *c-Myc*[38], as well as a potential therapeutic target. In addition, *FUBP1* has been shown to be associated with poor prognosis in glioma patients [33], and mutations in *CIC* and *FUBP1* have been reported to contribute to human oligodendroglioma [39]. Although *FUBP1* had clinical importance in various cancers, and was shown to be the top-ranked TMZ-related gene in our microarray data. When examining its prognostic value in the TCGA GBM cohort, *FUBP1* expression was found to have no significance in predicting the overall survival of GBM patients, which suggested *FUBP1* may not be an ideal biomarker for the prognosis of GBM patients.

*IDH1* mutation is a key molecular marker in WHO grades II and III gliomas [17]. It encodes the enzymes involved in cancer metabolism [40], and was initially described in diffuse glioma in 2008 [41]. *IDH1* mutations were present commonly in 70% of lower grade gliomas and secondary GBMs. However, they occurred at relatively lower frequency (5%) in primary GBMs [42] and in other malignancy [43, 44]. This finding was similar to our observation that there was only a small percentage of *IDH1* mutant in proneural subtype (13/60), neural subtype (1/43), and in all GBMs (14/248) (Supplementary Figure 1B). Therefore, this suggested that the *IDH1* mutant status may not be an ideal biomarker for predicting the survival of GBM patients due to the already existing high percentage of *IDH1* wild-type within primary GBMs, although the *IDH1* mutant status has been the most well-known prognostic and predicting biomarker for patients with lower grade gliomas. In our result, we also found *BICD1* expression was significantly correlated with *IDH1* expression, instead of the *IDH1* mutant status (Figure 11C), which suggested an emerging role of *BICD1* in *IDH1*-mediated cancer metabolism. In addition, the expression levels and expression status of *IDH1* were significantly higher in the classical and mesenchymal subtypes than in the proneural and neural subtypes of GBMs (Supplementary Figure 1C, 1D). This trend was similar with that in *BICD1* (Figure 3D, 3E).

*MGMT* is well-known for its repairing role in DNA damage induced by the alkylating agent TMZ, and has been identified as a powerful predicting biomarker for TMZ resistance. *MGMT* expression instead of *MGMT* promoter methylation was selected as a marker in this study because a straightforward correlation could be established between two different gene expressions. Another reason was a standard protocol to quantify the promoter methylation of *MGMT* is still lacking. Our analyses confirmed the well-known characteristic of *MGMT* expression in predicting patients’ response to TMZ treatment. Interestingly, we also found *MGMT* expression could significantly predict the response to radiation therapy, but not the overall survival of GBM patients.

Molecular classification of tumors is essential for developing personalized therapies. Since TMZ resistance is not mediated by only a single molecular event, but by multiple ones, using a multi-gene signature to predict the survival and therapeutic outcomes in cancer patients has become increasingly common recently. In this study, we stratified GBM patients into further distinct risk groups according to the combination of patient age, *MGMT* and *BICD1* expression to provide better prediction of patient’s prognosis and response to therapies, including TMZ and radiation. Although *MGMT* is a well-known biomarker for TMZ resistance in GBMs, combining *BICD1* with *MGMT* expression for risk stratification of GBM patients who received TMZ chemotherapy, may offer more therapeutic information for outcome assessment than *MGMT* alone.

Many cellular responses have been proposed to cause chemotherapeutic resistance in cancer cells, including apoptosis inhibition, DNA damage repair, drug target alteration, the epithelial-mesenchymal transition (EMT), drug efflux, and drug inactivation [45]. EMT is a process that controls the progressive loss of epithelial characteristics and the gain of mesenchymal features, the maintenance of cancer stemness, and the acquisition of chemoresistance [46]. The EMT spectrum, derived from cancer-specific transcriptomic EMT signatures for various cancers, is a method for universal scoring of EMT [47]. Our results showed that the expression levels of *BICD1* were significantly correlated with the EMT spectrum scoring (Figure 11A, 11B) and the expression levels of EMT markers in GBMs (Figure 12). This may suggest a possible connection between *BICD1* expression and EMT processing, which was associated with therapeutic resistance in cancers [48, 49].

*BICD1* was first reported to play a role in drosophila oocyte differentiation [50]. Another study group demonstrated that *BICD1* regulated G-protein signaling and internalization [51]. *BICD1* was also found to determine RNA binding and translational repression [52], and regulate the intracellular sorting and signaling of neurotrophin receptors [53]. An important study by Matanis *et al.*, published in *Nature Cell Biology* (2002), demonstrated that *BICD1* controls the coat complex coatomer protein I (COPI) independent Golgi-ER transport by recruiting the dynein-dynactin complex [54]. Dyneins are microtubule motors that are involved in many cellular processes, including mitosis and spindle assembly, nuclear migration and cell motility, and the transport of mRNA and a variety of cellular cargoes, including axonal and dendritic vesicles [55]. Another study suggested that the migration and proliferation of glioma cells correlate with high expression of cytoplasmic dynein and its regulatory proteins [56]. Wang *et al.* also reported that expression of dynein, cytoplasmic 2, heavy chain 1 (*DYNC2H1*) is associated with TMZ resistance in GBM cells [57]. Our analysis also showed a high correlation between the expression levels of *BICD1* and cytoplasmic dyneins (Supplementary Figure 2), which suggested another possible mechanism by which high *BICD1* expression may result in TMZ resistance in GBM cells through the dynein-mediated pathway.

In summary, our study indicated that high *BICD1* expression is associated with poor prognosis and therapeutic response in GBMs. Further investigation will be needed to explore the definite mechanism of *BICD1*-associated survival or therapeutic resistance in GBM cells by connecting with *IDH1*, EMT, or even dynein-mediated pathway, which may provide more understanding of the pathogenesis and therapeutic resistance in GBMs.

## MATERIALS AND METHODS

### Cell culture

Two human glioma cell lines (U87 and T98G) were maintained in DMEM (GIBCO, Grand Island, NY, USA). Mediums were all supplemented with 10% fetal bovine serum, penicillin (100unit/ml), and streptomycin (100μg/ml). Cells were incubated in 95% air, 5% CO_2_ humidified atmosphere at 37°C.

### MTT assay for cell viability analysis

U87MG and T98G cell viability were determined using MTT assays (Sigma Aldrich®). Cells were seeded in 96 well plates and add 20μl of 5mg/ml MTT at the end of the exposure time. The cells were incubated at 37°C for 4 hours, the medium was carefully removed, and 100μl of DMSO were added to each well. Absorbance was read at 570nm using an ELISA reader (Epoch, BioTek).

### Gene expression profiling from mRNA expression microarray

Total RNA extracted from cells with the A260/280 ratio greater than 1.9 was used in the Affymetrix microarray analysis. In the analysis, hybridization was performed by using Affymetrix human U133 2.0 plus arrays and the chips were scanned by Affymetrix GeneChip scanner 3000. Then, Affymetrix DAT files were processed by Affymetrix Gene Chip Operating System (GCOS) to generate CEL files. The raw intensities in CEL files were normalized by robust multi-chip analysis, and fold-change analysis was performed using GeneSpring GX11 (Agilent Technologies).

## RT-PCR

Equal amounts of total RNA were reverse transcribed into single-strand cDNA using the iScriptTM cDNA Synthesis Kit (Bio-RadTM). The mRNA was amplified using gene specific primers designed on the basis of available *BICD1* mRNAs conserved in the NCBI GenBank database. Each primer was check using the NCBI primer design tool (http://www.ncbi.nlm.nih.gov/tools/primer-blast/). The expression level of *BICD1* mRNAs were quantified relative to the expression level of the housekeeping gene, Glyceraldehyde-3-Phosphate-Dehydrogenase (*GAPDH*). The following primers were used:

*BICD1:* forward 5’-TGTTGAAAGCCAACA AGCAG-3’
and reverse 5’-TTGCAAACATTGCTCTCAGG-3’
(25 cycles, temperature of melting (Tm) is 50°C),
*GAPDH*: forward 5’-GAAGGTGAAGGTCGG AGT-3’
and reverse 5’-GAAGATGGTGATGGGATTTC-3’
(25 cycles, Tm is 60°C).

### Clinical data of glioma patients from the TCGA and CGGA websites

TCGA provides two distinct cohort databases of gliomas, including glioblastoma multiforme (GBM) cohort (grade IV glioma, n=523), and glioblastoma multiforme and lower grade glioma (GBMLGG) cohort (grades II, III and IV gliomas, n=689). All clinical data was downloaded from the TCGA Portal (http://www.xenabrowser.net/). In order to verify the consistency of results, we also collected clinical data in the Chinese Glioma Genome Atlas (CGGA) cohort from the CGGA Data Portal (http://cgga.org.cn/).

Patients’ clinical information, including gender, age, Karnofsky performance score (KPS), molecular subclassification, overall survival time, time to a new tumor event, time to tumor progression, time to tumor recurrence, and therapeutic type, were collected from the aforementioned website. Patients were split for survival analysis according to age and gender. For the age factor, a cut-off of 45 years was chosen in the TCGA GBMLGG cohort for approximately separating patients into two equal groups (age≤45, n=342 vs. age>45, n=347). And the age of 65 was determined in the TCGA GBM cohort because the definition for elderly is usually over 65 as in *NEJM* 2017 by Perry JR *et al.*[11] In each patient group, the overall survival, median survival time, 2 year-survival rate and HR, were obtained and compared using the Kaplan-Meier survival analysis.

### Genomic data of glioma patients from the TCGA and CGGA websites

The expression levels of candidate genes in the TCGA GBM cohort database (n=523) obtained by gene expression array (AffyU133a) and in the TCGA GBMLGG cohort database (n=689) obtained by gene expression RNAseq (polyA+ IlluminaHiSeq) were downloaded from the above TCGA Portal. For verifying the consistency of results, the gene expression profiling in the CGGA glioma cohort (n=220) obtained by mRNA expression microarray was also downloaded from the above CGGA Data Portal. Patients were split equally for survival analysis according to the expression status of *BICD1* and *MGMT.* Patients were also grouped according to the molecular subclassification of GBMs defined by TCGA [34] and the *IDH1* mutation status. In each patient group, the overall survival, median survival time, 2 year-survival rate and HR, were obtained and compared using the Kaplan-Meier survival analysis.

### Statistical analysis

All statistical analyses were performed using SPSS version 20.0 software (SPSS, Chicago, Illinois, USA). The unpaired *t-*test (the Student’s *t*-test) was used for analysis of the differences in gene expression between different subgroups of patients in the TCGA GBM and GBMLGG cohorts. Associations between *BICD1* expression and clinicopathological categorical variables were analyzed by Pearson’s Chi-square test. Estimates of the 2-year survival rate and survival curves were calculated using the Kaplan-Meier method, and differences in survival were compared by the log-rank test. To identify the factors that might have a significant influence on survival, univariate and multivariate analyses were performed using Cox’s proportional hazards regression modeling with and without adjustment for the expression status of candidate markers, age, gender, and molecular subclassifications, which were potentially related to survival. The scatter plot and box pictures were draw by using Prism 5 software (GraphPad software Inc.). For all analyses, a *P* value of <0.05 was considered statistically significant (ns: not significant; ^*^: *P*<0.05; ^**^: *P*<0.01; ^***^: *P*<0.001).

## CONCLUSION

There is an urgent need to identify biomarkers of GBMs to indicate patients’ prognosis and response to therapy. *BICD1* expression, a novel TMZ-related marker identified from GBM cell lines, may be a potential biomarker for prognosis and predicting the response to therapies, including TMZ and radiation therapies. However, the underlying mechanisms involved in *BICD1*-associated survival or therapeutic resistance in GBM cells, need further investigation.

## SUPPLEMENTARY MATERIALS FIGURES AND TABLES


