# Supplementary material for: BICD1 expression, as a potential biomarker for prognosis and predicting response to therapy in patients with glioblastomas
Source: Oncotarget. 2017 Nov 27;8(69):113766–91. doi: 10.18632/oncotarget.22667 (PMC5768362; doi:10.18632/oncotarget.22667)
Supplement: Supplementary file 1 [file oncotarget-08-113766-s001.pdf]

BICD1 expression, as a potential biomarker for prognosis and predicting response to therapy in patients with glioblastomas

SUPPLEMENTARY MATERIALS

A

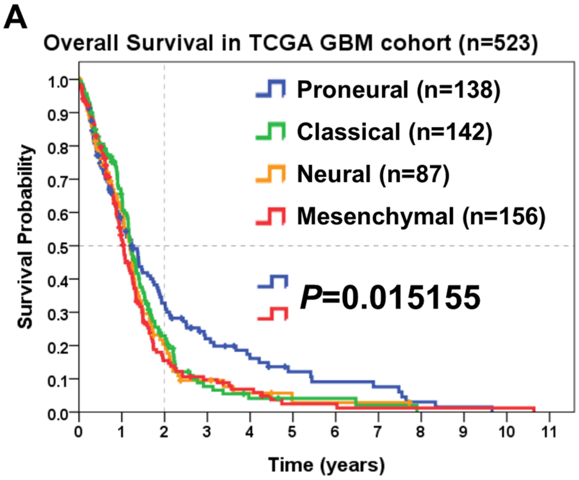

	Median survival	2-year survival	Adjusted HR	95% CI
Proneural	1.25	32.8%	-	-
Classical	1.21	22.9%	1.331	0.990-1.790
Neural	1.19	20.4%	1.239	0.951-1.613
Mesenchymal	1.05	15.5%	1.412	1.093-1.825
All	1.17	22.8%	-	-

(Proneural vs Classical: ns; Classical vs Neural: ns; Neural vs Mesenchymal: ns; Proneural vs Neural: ns; Proneural vs Mesenchymal: *)

GBM molecular subclassification		Proneural	Classical	Neural	Mesenchymal
Log Rank (Mantel-Cox)	Proneural		0.071523	0.089133	0.015155
	Classical	0.071523		0.590656	0.615182
	Neural	0.089133	0.590656		0.258565
	Mesenchymal	0.015155	0.615182	0.258565	

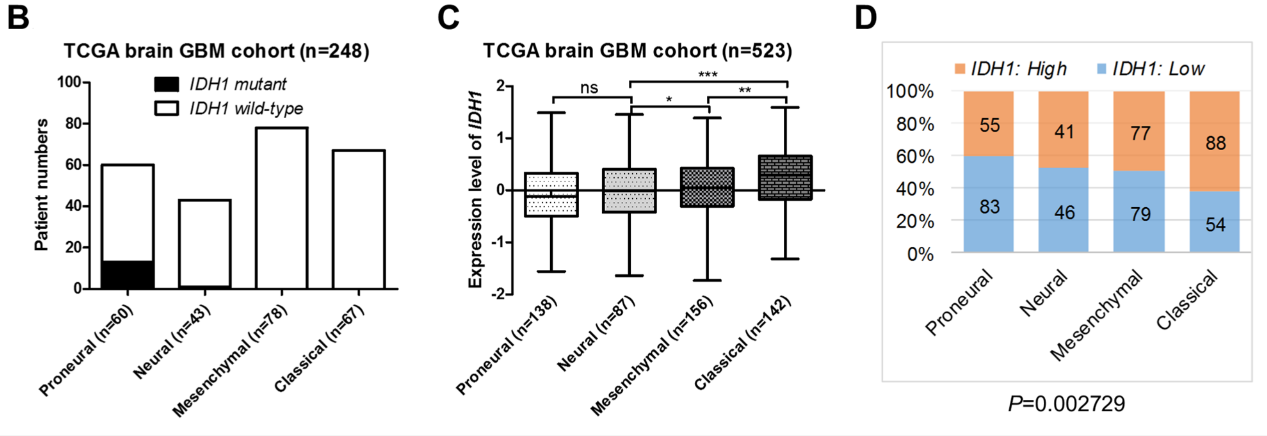

Supplementary Figure 1: Correlations between the *IDH1* status and the molecular subclassification of GBMs. (A) GBMs were classified into 4 distinct molecular subtypes according to the differentially expressed signature of 840 genes proposed by TCGA (Verhaak and the TCGA Research Network. Integrated Genomic Analysis Identifies Clinically Relevant Subtypes of Glioblastoma Characterized by Abnormalities in *PDGFRA, IDH1, EGFR,* and *NF1*. *Cancer Cell*, 2010). The proneural subtype had the best prognosis (median survival: 1.25 years, and 2-year survival rate: 32.8%). The mesenchymal subtype had the highest HR and the poorest prognosis (adjusted HR=1.412, median survival: 1.05 years, and 2-year survival rate: 15.5%) (*P*=0.015155). **(B)** *IDH1* mutations occurred mainly in the proneural subtype (13/60), rarely in the neural subtype (1/43), but were not found in the mesenchymal (0/78), and classical (0/67) subtypes of GBMs. **(C)** The expression levels of *IDH1* were significantly higher in the classical and mesenchymal subtypes than in the proneural and neural subtypes of GBMs (***). **(D)** The percentage of high *IDH1* expression was significantly higher in the classical and mesenchymal subtypes than in the proneural and neural subtypes of GBMs (*P*=0.002729).

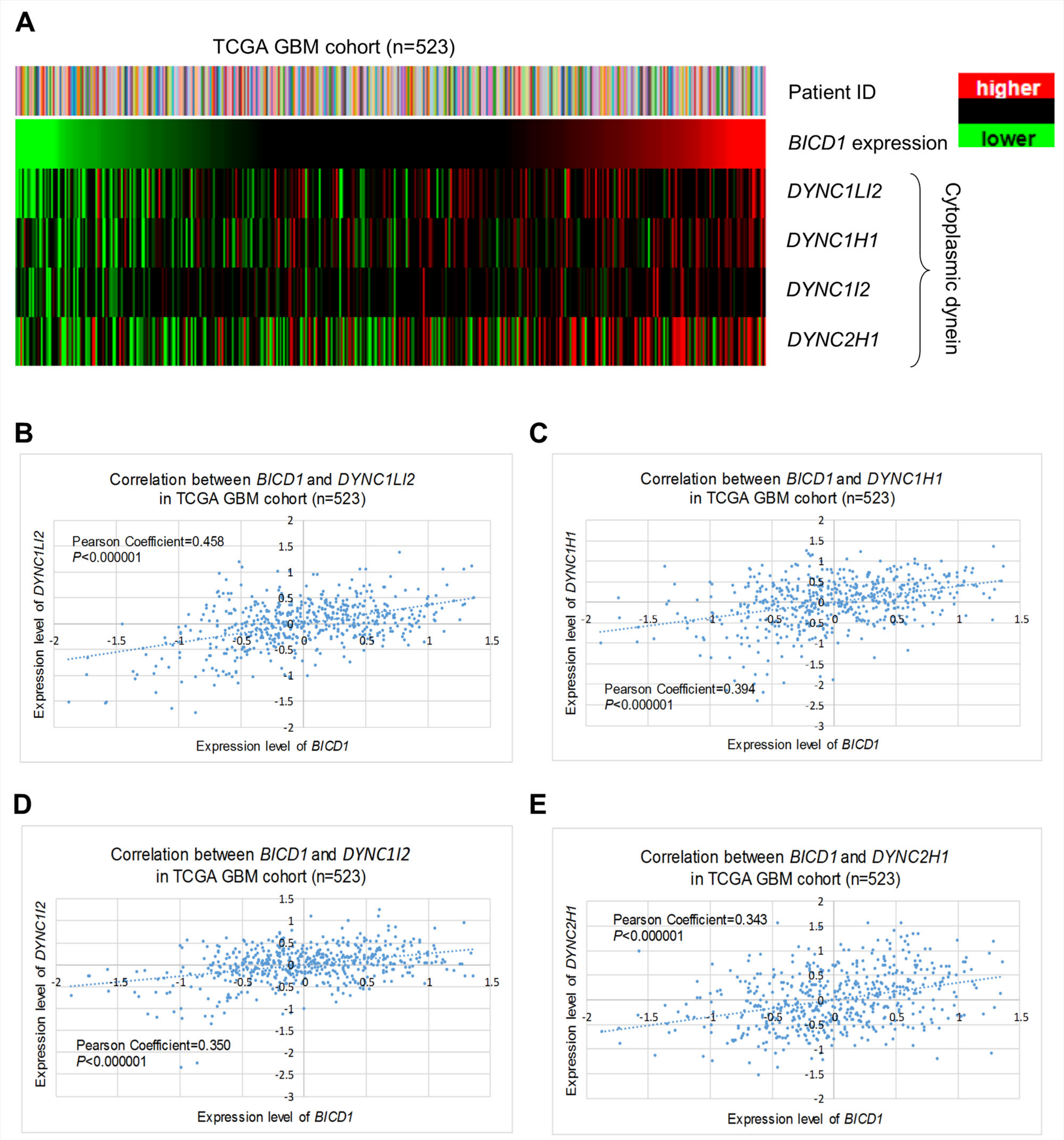

Supplementary Figure 2: Correlations between expression of *BICD1* and cytoplasmic dyneins. (A) *BICD1* expression appeared to be positively correlated with expression of cytoplasmic dyneins (*DYNC1LI2, DYNC1H1, DYNC1I2,* and *DYNC2H1*) in the TCGA GBM cohort (n=523). **(B)** *BICD1* expression was significantly and positively correlated with *DYNC1LI2* expression ($P<0.000001$, Pearson's correlation coefficient=0.458). **(C)** *BICD1* expression was significantly and positively correlated with *DYNC1H1* expression ($P<0.000001$, Pearson's correlation coefficient=0.394). **(D)** *BICD1* expression was significantly and positively correlated with *DYNC1I2* expression ($P<0.000001$, Pearson's correlation coefficient=0.350). **(E)** *BICD1* expression was significantly and positively correlated with *DYNC2H1* expression ($P<0.000001$, Pearson's correlation coefficient=0.343).

Supplementary Table 1: Correlation of *BICD1* expression with the KPS of patients in the TCGA GBM cohort

Clinicopathological feature	n	*BICD1* expression, n (%)		*P*
	393	Low, n=197 (50.1)	High, n=196 (49.9)	
**KPS**				0.609816
≥90	68	36 (52.9)	32 (47.1)	
<90	325	161 (49.5)	164 (50.5)	

KPS: Karnofsky performance score.

Supplementary Table 2: The overall survival of patients in different molecular subtype of GBMs

Molecular subtype	Patient numbers	Death event numbers	Median value				5-year survival rate	2-year survival rate
			Survival time (years)	S.D.	Lower limit (95% CI)	Upper limit (95% CI)		
**Proneural**	138	110	1.247	0.146	0.960	1.533	12.1%	32.8%
**Classical**	142	117	1.208	0.062	1.086	1.330	4.1%	22.9%
**Neural**	87	75	1.192	0.090	1.016	1.368	2.9%	20.4%
**Mesenchymal**	156	132	1.047	0.054	0.941	1.152	2.5%	15.5%
**All**	523	434	1.17	0.044	1.083	1.257	5.5%	22.8%
